# Role of PSIP1/LEDGF/p75 in Lentiviral Infectivity and Integration Targeting

**DOI:** 10.1371/journal.pone.0001340

**Published:** 2007-12-19

**Authors:** Heather M. Marshall, Keshet Ronen, Charles Berry, Manuel Llano, Heidi Sutherland, Dyana Saenz, Wendy Bickmore, Eric Poeschla, Frederic D. Bushman

**Affiliations:** 1 Department of Microbiology, University of Pennsylvania School of Medicine, Philadelphia, Pennsylvania, United States of America; 2 Department of Family, Preventive Medicine, San Diego School of Medicine, University of California at San Diego, San Diego, California, United States of America; 3 Medical Research Council (MRC) Human Genetics Unit, Edinburgh, United Kingdom; 4 Molecular Medicine Program, Mayo Clinic College of Medicine, Rochester, Minnesota, United States of America; The Research Institute for Children, United States of America

## Abstract

**Background:**

To replicate, lentiviruses such as HIV must integrate DNA copies of their RNA genomes into host cell chromosomes. Lentiviral integration is favored in active transcription units, which allows efficient viral gene expression after integration, but the mechanisms directing integration targeting are incompletely understood. A cellular protein, PSIP1/LEDGF/p75, binds tightly to the lentiviral-encoded integrase protein (IN), and has been reported to be important for HIV infectivity and integration targeting.

**Methodology:**

Here we report studies of lentiviral integration targeting in 1) human cells with intensified RNAi knockdowns of PSIP1/LEDGF/p75, and 2) murine cells with homozygous gene trap mutations in the *PSIP1/LEDGF/p75* locus. Infections with vectors derived from equine infections anemia virus (EIAV) and HIV were compared. Integration acceptor sites were analyzed by DNA bar coding and pyrosequencing.

**Conclusions/Significance:**

In both PSIP1/LEDGF/p75-depleted cell lines, reductions were seen in lentiviral infectivity compared to controls. For the human cells, integration was reduced in transcription units in the knockdowns, and this reduction was greater than in our previous studies of human cells less completely depleted for PSIP1/LEDGF/p75. For the homozygous mutant mouse cells, similar reductions in integration in transcription units were seen, paralleling a previous study of a different mutant mouse line. Integration did not become random, however–integration in transcription units in both cell types was still favored, though to a reduced degree. New trends also appeared, including favored integration near CpG islands. In addition, we carried out a bioinformatic study of 15 HIV integration site data sets in different cell types, which showed that the frequency of integration in transcription units was correlated with the cell-type specific levels of *PSIP1/LEDGF/p75* expression.

## Introduction

Early steps of retroviral replication involve reverse transcription to generate a DNA copy of the viral RNA genome, and integration, which results in the covalent connection of the viral DNA to host cell DNA (for reviews see [1], [2]). The question of where retroviruses target DNA integration is central to understanding viral host interactions. For the virus, selection of favorable sites for viral DNA integration assists efficient expression of the viral genome after integration [3]–[6]. For the host, viral DNA integration can either activate or inactivate gene transcription. One consequence of integration can be insertional activation of oncogenes and transformation to malignant growth [1], [2], [7], [8]. Here we present data on the role of a host-cell encoded protein, PSIP1/LEDGF/p75, that guides integration site selection by lentiviruses, the viral genus including HIV (henceforth we use “LEDGF/p75” because this name is widely used in the HIV field).

LEDGF/p75 first came to the attention of the retrovirus field when it was identified in affinity-based screens for its tight binding to HIV IN [9]–[11]. LEDGF/p75 tethers ectopically-expressed HIV IN to chromatin [9], [10], [12], [13], through specific binding domains [14]–[17], and also protects IN from proteasomal degradation [18]. LEDGF/p75 binding is specific for lentiviral IN proteins (e. g. those of HIV, SIV, FIV, and EIAV)[12], [19], [20], which makes it appealing as a candidate tethering factor since all the lentiviruses tested (HIV, SIV, FIV, and EIAV) show favored integration in active transcription units [5], [21]–[32]. The crystal structure of the catalytic domain of HIV IN (residues 50–212) bound to the integrase binding domain (IBD) was solved, which showed that a pair of LEDGF/p75-IBD molecules could bind at symmetry-related positions at the interface of the IN catalytic domain dimer [33], [34]

Early attempts to determine whether LEDGF/p75 was important for efficient HIV replication used RNAi knockdowns in human cells, which had either no effect or quantitatively modest effects on infection [12], [13], [35], [36]. This now appears to be because incomplete knockdowns left biologically significant amounts of protein present. More recently, human SupT1 cells with intensified RNAi knockdowns showed drops of 30-fold for infection by either HIV or another lentivirus, feline immunodeficiency virus (FIV), and combining this with dominant interfering proteins derived from the LEDGF/p75-IBD produced 560-fold inhibition of infection [37]. These findings are supported by additional studies in human cell lines [35], [38], [39].

Early knockdowns of LEDGF/p75 were also analyzed for effects on targeting of HIV integration [40]. Knockdowns in three cell types were studied, and in each integration frequency within transcription units was reduced. In addition, other effects were seen, including an increase in the content of G/C bases around sites of HIV integration in the knockdown cells. These data supported the idea that LEDGF/p75 acted as a tethering factor, binding to both HIV and chromatin to direct HIV integration into active genes. In support of the tethering model, artificial fusion proteins in which the LEDGF/p75 IBD was fused to the sequence specific DNA binding domain of phage lambda repressor were shown to direct favored integration *in vitro* near repressor binding sites [24]. Also supporting the tethering idea, function of LEDGF/p75 in promoting HIV replication requires that both ends of the putative LEDGF/p75 tether be intact [37].

However, key questions still remain on the role of LEDGF/p75. In all the models studied, HIV continued to favor integration within active transcription units. This could either be because residual LEDGF/p75 remaining in the knockdown was sufficient for residual targeting activity, or because additional host cell factors also contribute independently to targeting HIV integration. In an effort to address this issue, Shun et al. prepared a mouse strain in which part of the *LEDGF/p75* locus was flanked by Cre recombination sites [41], and the *LEDGF/p75* exon was deleted by exposure to Cre recombinase. Mouse embryonic fibroblasts were then studied for effects on infection with HIV reporter viruses. These cells showed a 20-fold reduction in infectivity by HIV, and also a reduction in integration frequency in transcription units that was stronger than that reported in human cell knockdowns by Ciuffi et al. [40]. However, HIV did still infect at a reduced rate, and integration in transcription units was still significantly favored. The mouse cells also showed some new targeting features in the LEDGF/p75-depleted cells, including increased integration near CpG islands.

These studies were helpful in clarifying the effects of strong LEDGF/p75 depletion, but several issues remain. We wished to obtain lentiviral integration targeting data for human cells with stronger knockdowns of LEDGF/p75 to investigate possible effects of the host cell species. We also wished to obtained data from an additional murine cell line depleted for LEDGF/p75 to check the generality of conclusions from Shun et al. [41]. We thus studied the human SupT1 T-cell line with intensified RNAi developed by Llano et al. [37], and mouse cells containing homozygous gene trap mutations at the *LEDGF/p75* locus developed by Sutherland and coworkers [42]. Vectors derived from equine infectious anemia virus (EIAV) were used in many of the experiments, allowing effects on HIV and EIAV to be compared. Studies of both cell models and both lentiviruses provided strong evidence for the role of LEDGF/p75 in promoting efficient infection and targeting integration in transcription units. In addition to these data on manipulated cell models, we also present additional bioinformatic studies of 15 published HIV integration site data sets in different cell types, which revealed a strong correlation between cell type specific *LEDGF/p75* expression levels and the proportion of HIV integration sites in transcription units. These data provide further support for the generality of LEDGF/p75 as a determinant of integration target site selection for lentiviruses in primary cells where LEDGF/p75 levels were not artificially reduced.

## Results

### Efficiency of lentivirus infection in human SupT1 cells with intensified knockdown of LEDGF/p75

Initially cells depleted for LEDGF/p75 were tested for effects on lentiviral infection. For the human SupT1 cells with the intensified LEDGF/p75 knockdown (the TC2 and TL2 cell lines in [37]), there were technical complications in studying HIV integration targeting. To generate the cells, shRNAs were introduced using HIV-based vectors. Thus the modified cells already contain integrated HIV sequences, which would complicate sequence analysis of newly integrated HIV proviruses. For that reason, we studied the lentivirus equine infectious anemia virus (EIAV). Like HIV IN, EIAV IN is known to bind LEDGF/p75 [20], and EIAV is also known to integrate in active transcription units [31], so EIAV is a suitable model for analysis of the influence of LEDGF/p75 on lentivirus infection.

**Figure 1:**
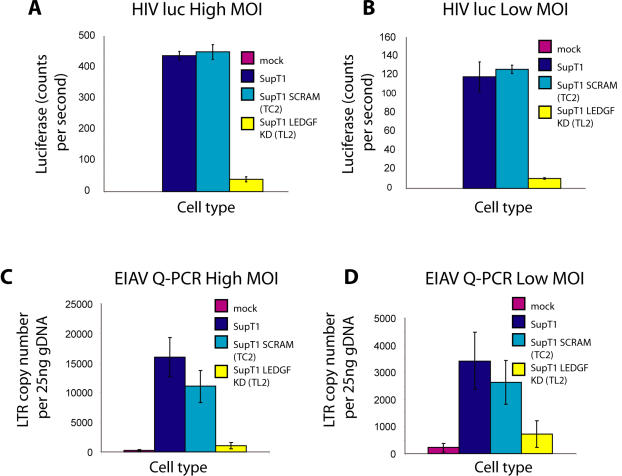
Effects of intensified knockdown of LEDGF/p75 in SupT1 cells on the efficiency of lentiviral infection. A) and B) HIV luc activity was compared for wild-type SupT1 cells, SupT1 containing a control scrambled shRNA (SCRAM), and LEDGF/p75 knockdown (KD) cells. A) High multiplicity of infection (80 ng p24); B) Lower multiplicity of infection (20 ng p24). The designation “p24” indicates the amount of viral stock, measured by the weight of the p24 capsid antigen applied to cells. C) and D) EIAV infectivity was compared in the SupT1 cell set as assayed by quantitative PCR for viral cDNA: C) high multiplicity (100 µl stock), D) lower multiplicity (25 µl stock).

Figure 1 shows the efficiency of infection by HIV and EIAV in the modified SupT1 cells. HIV infection efficiency was characterized two days after infection using a luciferase-transducing HIV vector. Activity was compared for unmodified SupT1 cells or control cells containing a scrambled shRNA sequence (SCRAM). Luciferase activity was reduced ∼10 fold in the LEDGF/p75 knockdown but not in the control cells, and similar effects were seen at two multiplicities of infection (Figure 1A and B), paralleling previously published data from Llano et al. [37].

An EIAV vector was also tested (Figure 1C and D). Infection through the step of integration was monitored by infecting cells, then growing the cells for two weeks, so that only covalently integrated DNA persisted (unintegrated DNA is degraded or lost by dilution during prolonged cell growth [43], [44]). EIAV DNA was then quantified in genomic DNA samples using quantitative PCR. The LEDGF/p75 knockdown cells showed only between 8 and 24% of the amount of viral DNA seen in the control cells, indicating that for EIAV as well LEDGF/p75 is important for completing the early steps of replication.

### Efficiency of lentivirus infection in murine cells disrupted at *LEDGF/p75*

We also compared lentiviral infection in murine cells containing the gene trap disruption of *LEDGF/p75* reported by Sutherland and colleagues [42]. Because residual expression is sometimes detected in gene trap alleles, we used quantitative RT-PCR to determine the fraction of *LEDGF/p75* messages disrupted by the gene trap insertion. In samples from homozygous mutant (−/−) cells, amplification of correct *LEDGF/p75* message was sporadically detected at high PCR cycle numbers, suggesting that rare correctly spliced messages were formed. However, quantification of correct message formation using SyberGreen quantitative PCR showed expression of *LEDGF/p75* to be below the limit of detection in the −/− cells, corresponding to a reduction of at least 32-fold compared to the wild type (+/+) cells (unpublished data). Sutherland and coworkers reported LEDGF/p75 protein to be undetectable [42].

**Figure 2:**
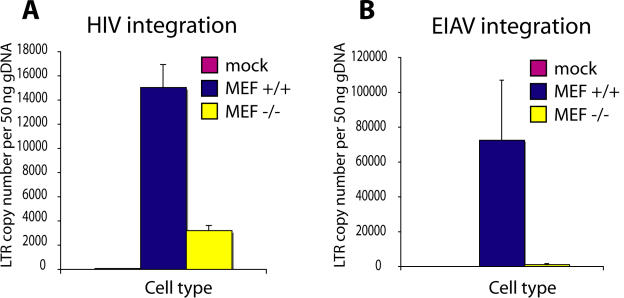
Efficiency of lentiviral infection in control (+/+) and homozygous *LEDGF/p75*-disrupted (−/−) murine cells, measured by quantitative PCR. A) HIV infectivity. B) EIAV infectivity.

We analyzed infection of murine embryonic fibroblasts (MEFs) isolated from embryos of +/+ and homozygous mutant −/− mice after infection with HIV and EIAV. Integration was measured by infecting cells, maintaining the cells in culture for two weeks to allow loss of unintegrated DNA [44], then quantifying the viral DNA by TaqMan PCR. HIV integration was reduced ∼five fold in the *LEDGF/p75* −/− MEFs (Figure 2A), and EIAV integration was reduced >50 fold. Thus in the presence of a homozygous mutation of *LEDGF/p75*, lentiviral integration was strongly reduced but not eliminated.

### DNA bar coding and pyrosequencing to analyze integration site placement

Below we first describe studies of EIAV integration targeting in the SupT1 cells with intensified RNAi knockdowns, then HIV and EIAV targeting in the mouse cells disrupted at *LEDGF/p75*.

For each of our studies, we used the pyrosequencing technology commercialized by 454 Life Sciences [45] to sequence genomic DNA flanking integrated proviruses. Briefly, genomic DNA was isolated and cleaved with restriction enzymes. DNA linkers were ligated onto the cleaved ends, then host-virus DNA junctions were amplified using one primer complementary to the linker and one complementary to the viral DNA end. A second round of PCR was used to improve specificity and to add recognition sites for the 454 primers necessary for the emulsion PCR step preceding pyrosequencing [46]. Pooled DNAs were then subjected to pyrosequencing.

Use of DNA bar coding allowed multiple integration site populations to be studied in parallel [47]–[49]. The viral DNA primer used in the second round of amplification contained a short recognition sequence (4–8 bases) abutting the 454 primer that was different for each sample tested. These 4–8 bases are the first determined in pyrosequencing reads. Thus use of bar coding allowed many samples to be pooled for sequence determination, then the reads could be sorted into individual experiments by bar code. A total of 3566 unique integration site sequences from different virus and cell combinations were determined using this method (Table 1).

**Table 1:** Integration site data sets used in this study.

Cell line	Description and LEDGF/p75 status	Virus	Number of Integration Sites	Source of sequences analyzed
SupT1	Human SupT1 cell line	EIAV vector	783	This report
TC2 (SupT1 SCRAM)	Human SupT1 cell line with ilvRNAi with scramble shRNA, polyclonal	EIAV vector	869	This report
TL2 (SupT1 LEDGF KD)	Human SupT1 cell line with ilvRNAi for LEDGF/p75, polyclonal	EIAV vector	157	This report
Jurkat siJK2	Human Jurkat cell line, shRNA LEDGF/p75 knock down	HIV vector	695	[37]
Jurkat-siJK2BC	Human Jurkat cell line, shRNA LEDGF/p75 knock down back complimented with p75/LEDGF insensitive to the shRNA	HIV vector	685	[37]
293T-siLL	Human 293T cell line, with shRNA LEDGF knock down	HIV vector	593	[37]
293T-siScram	Human 293T cell line, with scramble shRNA	HIV vector	450	[37]
iMEF +/+	Murine embryonic fibroblasts from wild-type mice (immortalized)	HIV vector	574	This report
iMEF −/−	Murine embryonic fibroblasts from knockout mice (immortalized)	HIV vector	287	This report
prMEF +/+	Murine embryonic fibroblasts from wild-type mice (primary)	HIV vector	531	This report
prMEF −/−	Murine embryonic fibroblasts from knockout mice (primary)	HIV vector	209	This report
iMEF +/+	Murine embryonic fibroblasts from wild-type mice (immortalized)	EIAV vector	70	This report
iMEF −/−	Murine embryonic fibroblasts from knockout mice (immortalized)	EIAV vector	86	This report

### Consensus sequences at EIAV integration sites in human SupT1 cells

The EIAV vector was used to infect SupT1 cells with intensified RNAi against LEDGF/p75 and compared to controls consisting of either SupT1 cells with a scrambled shRNA (SCRAM) or untreated SupT1 cells. Integration sites were sequenced and placed on the hg18 draft human genome sequence. As a first step in the analysis, the favored target DNA sequences at the point of integration were compared in the presence and absence of LEDGF/p75. Alignment of target DNA sequences at integration sites has revealed weak inverted repeat consensus sequences [50]–[55], the symmetry arising because the favored sequence features at each of the two viral DNA ends are the same. The presence of this consensus sequence can be a strong predictor of integration targeting specificity, particularly over short intervals [55]. For HIV, the favored consensus sequence has been synthesized and shown to be a favored integration target site for HIV preintegration complexes *in vitro* [52].

**Figure 3:**
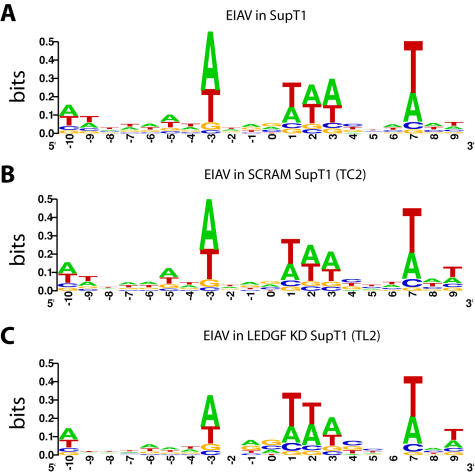
Integration site consensus at sequences flanking EIAV proviruses in control and LEDGF/p75-knockdown SupT1 cells. A) Unmodified SupT1 cells. B) Control SCRAM cells. C) LEDGF KD cells. The diagrams were generated using the WebLOGO program (weblogo.berkeley.edu/logo.cgi). The y-axis indicates bits of information–perfect conservation of a base would score as two bits.

EIAV has been reported to favor integration in an A/T rich palindromic consensus sequence [31], which matched that seen here for EIAV integration in the control SupT1 and SupT1 SCRAM cell lines (Figure 3A and B). The LEDGF/p75 knockdown cells showed an indistinguishable consensus sequence (Figure 3C), providing evidence against the view that LEDGF/p75 is involved in specifying the target sequence preference.

### EIAV integration targeting in human SupT1 cells depleted for LEDGF/p75

**Figure 4:**
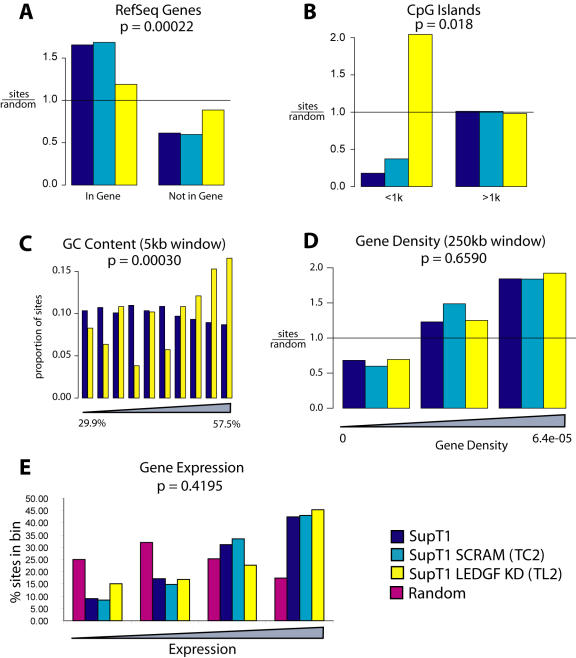
EIAV integration site distributions in control and LEDGF/p75-knockdown SupT1 cells. Integration site distributions are shown relative to A) RefSeq genes, B) CpG islands (plus or minus 1 kb), C) relative G/C content (Integration sites from unmodified and knockdown cells were pooled and divided into 10 equal bins of increasing GC content, and sites in each cell type plotted for each bin), D) gene density, and E) relative gene expression intensity. For each value in A–B) and D), the measured value for the integration site population was divided by that of the matched random control to emphasize the departure of the experimental data from random. P values shown are based on regression analysis (A–C) or Chi Square test for trend (D–E).

The genomic distribution of EIAV integration sites was then compared in the presence and absence of LEDGF/p75 (Table 2 and Figure 4). Integration site data sets were compared systematically relative to many forms of genomic annotation. Complete reports are in Statistical Reports S1 and S2. Major findings are summarized below.

**Table 2:** Integration frequency in the presence and absence of LEDGF/p75 near mapped genomic features in the human genome.

	Frequency in Genomic Feature (%)				
	Transcription Units				
Data Set	Known	RefSeq	Unigenes	<2 kb CpG Island	<5 kb Gene 5′ End
EIAV in SupT1	66.5***	60.3***	59.6***	0.64**	6.5
EIAV in TC2 (SCRAM) SupT1	69.4***	61.8***	61.9***	0.92**	5.4
EIAV in TL2 (LEDGF KD) SupT1	50.3	44.6*	51.6*	5.7	9.6
Random Control	43.3	36.5	42.2	2.4	5.6

Significant deviation from matched random controls according to the Fisher's exact test is denoted by * (***p<0.0001, **p<0.01, *p<0.05). The ‘random control’ set is the matched random control set for the SupT1 integration set (see Materials and Methods for generation of matched random controls).

Three catalogs of human gene annotation were used to analyze EIAV integration site distributions, since LEDGF/p75 had previously been implicated in directing HIV integration to transcription units. From 60 to 69% of EIAV integration sites were in genes (Table 2), while a computationally generated random distribution showed only 37 to 43 % in genes. In the cell line strongly depleted for LEDGF/p75, integration frequency in genes ranged from 45% to 52%, a significant reduction compared to the pooled SupT1 and SupT1 SCRAM controls (P<0.0001 for Known genes, P<0.0001 for RefSeq, P = 0.027 for Unigenes; comparison to pooled controls by the Fisher's exact test). However, even in the absence of LEDGF/p75, integration in genes was still significantly favored over random in two out of three sets of gene calls (Table 2). Figure 4A shows the extent of favoring of integration in RefSeq transcription units normalized to the random expectation.

In some data sets integration by lentiviruses has been found to be disfavored near CpG islands [22], which are genomic regions enriched in the rare CpG dinucleotide and commonly associated with transcription start sites and regulatory regions. EIAV also showed disfavored integration near CpG islands (P<0.0001 for comparison random sites by the Fisher's exact test). In the LEDGF/p75-depleted cells, integration frequency within 2 Kb of CpG islands went up, so that CpG islands were no longer disfavored (Table 2), and the difference between pooled SupT1 control sites and LEDGF/p75-depleted cells achieved significance (P<0.0001, Fisher's exact test). Figure 4B shows the frequency within 1 kb, plotted to emphasize the enrichment over random.

CpG islands are often associated with transcription start sites. Analysis of integration frequency showed a trend toward more frequent integration near transcription start sites in the knockdown (6% in pooled SupT1 controls versus 10% in the knockdown) though the trend did not achieve significance with this sample size (P = 0.083 by the Fisher's exact test).

In the previous study of weaker LEDGF/p75-knockdowns [40], HIV integration in knockdown cells was associated with an increase in the relative G/C content at integration sites. One speculation was that this was because LEDGF/p75 contains an A/T hook DNA binding domain, which may promote integration in A/T-rich regions in LEDGF/p75-positive cells [40]. Figure 4C shows that in the SupT1 cell model as well, strong depletion of LEDGF/p75 resulted in increased G/C content at integration sites (P = 0.0003 by regression analysis).

**Figure 5:**
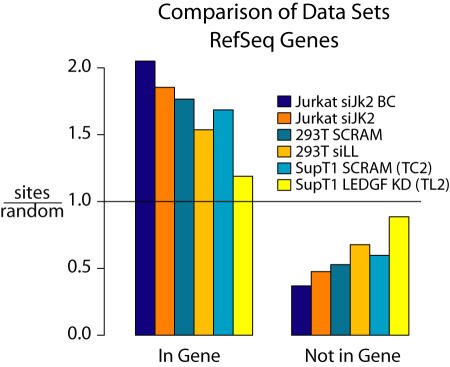
Comparison of LEDGF/p75 knockdowns in different human cell types. The Jurkat and 293T data sets are described in detail in [40]. Integration frequency was compared within RefSeq genes.

One of the main questions at the start of this study was whether a stronger knockdown of LEDGF/p75 would result in stronger effects on lentivirus integration targeting. Figure 5 shows a comparison of two HIV integration site data sets from Ciuffi et al. for HIV integration in Jurkat or 293T cells [40], which harbored less complete knockdowns of LEDGF/p75. In the control cells (Figure 5, blue shading) integration was enriched in transcription units in all cases. In the LEDGF/p75 knockdowns (Figure 5, orange and yellow shading), the proportion of integration sites in genes was reduced, with the percent change significantly greater in the intensified SupT1 knockdown over many of the gene catalogs studied.

Integration frequency at some of the genomic features studied was not detectably affected by the LEDGF/p75 knockdown. For example, when integration frequency was assessed relative to gene density, no strong effect was seen (Figure 4D). Similarly, the relationship between gene activity and integration frequency was not significantly altered (Figure 4E). Integration frequency near open chromatin as marked by DNAse I hypersensitive sites was also not significantly altered by the knockdown (data not shown). This implies that either there is residual LEDGF/p75 present even in the intensified knockdowns that is sufficient to influence targeting, or else other cellular systems contribute to integration targeting as well.

### Consensus sequences at lentiviral integration sites in murine cells disrupted at *LEDGF/p75*

We analyzed integration sites in murine embryonic fibroblasts (MEFs) derived from the *LEDGF/p75* homozygous gene trap (−/−) and control (+/+) mice [42] after infection with HIV and EIAV. Cells that had been immortalized in culture (iMEF) were compared to primary MEFs (prMEFs). For all the features discussed below the results were identical for iMEFs and prMEFs (data not shown), so the two data sets were pooled in what follows.

**Figure 6:**
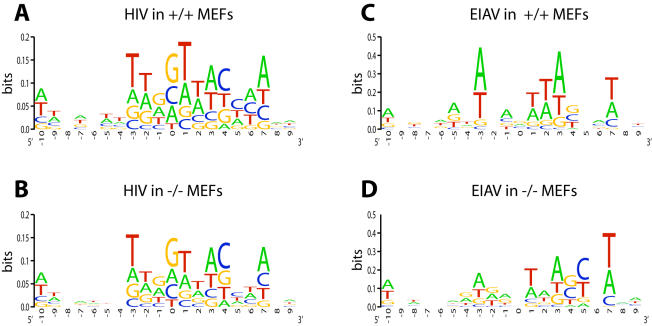
Integration site consensus sequence for lentiviral infection of murine control and *LEDGF/p75*-disrupted cells. A) HIV in +/+ MEFs. B) HIV integration in −/− MEFs. C) EIAV integration in +/+ MEFs. D) EIAV integration in −/− MEFs. Markings as in Figure 3.

Integration site sequences were aligned to determine the consensus palindromic sequence at the point of integration, and results were compared for the +/+ and −/− MEFs for each virus (Figure 6). In both cases, integration in the +/+ MEFs showed the weak consensus seen previously for HIV and EIAV. No major differences were seen in the −/− MEFs, consistent with findings described above for human cells and previously [40], [41].

### EIAV integration targeting in murine cells disrupted at *LEDGF/p75*

Genome-wide studies of EIAV integration targeting in murine cells are presented in this section and analysis of HIV integration in murine cells is described in the next section. Extensive further analysis of EIAV and HIV integration in MEFs is presented in Statistical Reports S2.

**Figure 7:**
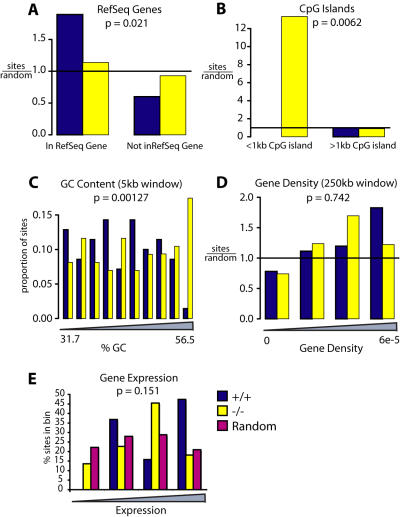
EIAV integration distributions in murine control and *LEDGF/p75*-disrupted cells. Integration frequencies are shown relative to A) RefSeq genes, B) CpG islands (1 kb window; note that there were no control sites within <1 kb), C) G/C content, D) Gene density (250 kb window), E) Gene activity. Markings as in Figure 4.

EIAV integration in transcription units was decreased in the −/− *LEDGF/p75* gene trap cells compared with wild-type. In wild-type cells, 58.6% of experimental integration sites were in RefSeq genes (see Table 3), a significant enrichment over the 28% seen in the matched random controls (see Figure 7A). In −/− MEFs, 38.4% of sites were in RefSeq transcription units, a value that is significantly less than in the +/+ MEFs (p = 0.016 by the Fisher's exact test). Significant differences were seen when the analysis was repeated using other gene catalogs as well (Table 3).

**Table 3:** Integration frequency in the presence and absence of LEDGF/p75 near mapped genomic features in the murine genome.

	Frequency in Genomic Feature (%)				
	Transcription Units				
Data Set	Known	RefSeq	Ensemble	<2 kb CpG Island	<5 kb Gene 5′ End
HIV in +/+ MEF	58.6***	54.3***	60.7***	0.7*	10.9***
HIV in −/− MEF	42.9***	38.7***	46.0***	6.5***	15.5***
EIAV in +/+ MEF	62.9***	58.6***	64.3***	1.4	5.7
EIAV in −/− MEF	41.9	38.4	45.3	12.8***	25.6***
Random Control	29.7	28	32	1.7	6.8

Significant deviation from matched random controls according to the Fisher's exact test is denoted by * (***p<0.0001, **p<0.01, *p<0.05). The ‘random control’ set shown is the matched random control set for the HIV +/+ integration set (see Materials and Methods for generation of matched random controls).

We also analyzed the proximity of EIAV integration sites to CpG islands (Figure 7B and Table 3). In wild-type cells integration within 2 kb of CpG islands was not significantly different from random, while in knockout cells integration was 13-fold enriched over random (P = 0.0086; Fisher's exact test).

The frequency of integration within 5 kb of RefSeq gene 5′ ends showed a similar pattern (Table 3). Integration levels around gene 5′ ends were not significantly different from random in the +/+ cells (5.7% of sites), whereas in the knockout a significant enrichment was observed (25.6% of sites) achieving P = 0.014 for the comparison between cell types (Fisher's exact test).

We analyzed the correlation between integration frequency and G/C content using a 5 kb window around the integration site. A significant difference between genotypes was found (P = 0.001, using regression analysis, Figure 7C).

A variety of features analyzed did not show significant differences between genotypes, including the response to gene density (Figure 7D) and the relationship between gene activity and integration frequency (Figure 7E). We return to the implications of these findings in the Discussion.

### HIV integration targeting in murine cells disrupted at *LEDGF/p75*

**Figure 8:**
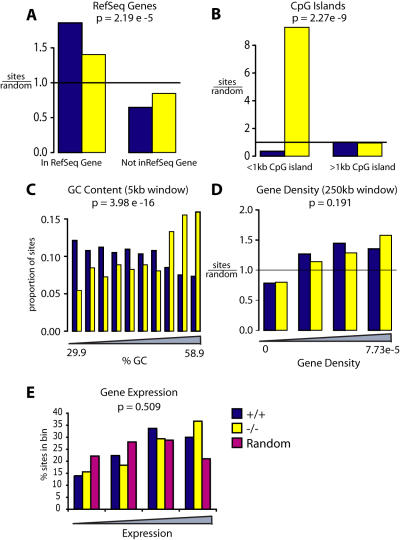
HIV integration distributions in murine control and *LEDGF/p75*-disrupted cells. A) RefSeq genes, B) CpG islands (1 kb window), C) G/C content, D) Gene density (250 kb window), E) Gene activity. Markings as in Figure 4.

Data on HIV integration site distributions in MEFs closely matched the data for EIAV integration (Figure 8 and Table 3). HIV integration in +/+ MEFs showed a strong preference for transcription units (Table 3 and Figure 8), which was strongly reduced in the −/− MEFs (P<0.0001 for comparison between genotypes).

HIV integration within 2 kb of CpG islands was found to be disfavored compared with matched random controls, and this was the case in +/+ MEFs (Figure 8B and Table 3). Integration in −/− MEFs was greatly increased within 2 kb of CpG islands or 5 kb of transcription start sites (P<0.0001 and P = 0.014 or the respective comparisons between genotypes).

Knockdown of LEDGF/p75 has previously been shown to result in an increase in the G/C content of HIV integration site sequences [40]. We therefore analyzed the frequency of integration in regions of varying G/C content (Figure 8C), revealing that integration was significantly increased in more G/C rich regions in the −/− MEFs (P = 4e-16).

As seen above for EIAV, the frequency of integration near a variety of features was not detectably altered. Figure 8D shows that integration frequency was similarly favored in gene-rich regions in both the +/+ and −/− MEFs. Figure 8E shows that the relative activity of genes hosting integration events was also not distinguishable for the +/+ and −/− MEFs (Figure 8E).

### Correlation between *LEDGF/p75* expression and the frequency of HIV integration in transcription units analyzed over many cell types

In addition to studying cells with artificially reduced levels of *LEDGF/p75* expression, we were interested in natural variation in cellular *LEDGF/p75* expression levels. Different primary cell types and cell lines show different steady state levels of *LEDGF/p75* mRNA. Different cell types also show reproducibly different frequencies of HIV integration in transcription units (see [40] for examples). We thus asked whether cell types with higher LEDGF/p75 levels showed higher frequencies of HIV integration transcription units.

**Figure 9:**
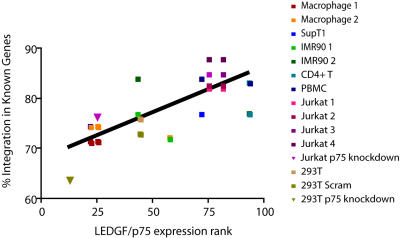
Correlation between LEDGF/p75 expression and the frequency of HIV integration in genes. Data is shown for 15 HIV integration site data sets in 10 cell types. The y-axis shows the percentage of integration events within transcription units of the “known gene” set of human genes for each integration site data set. The x-axis shows relative expression values for *LEDGF/p75* derived from Affymetrix array data (see methods for details). The R-squared value for the fit is 0.6148 (P<0.0001). The references for the data sets used are as follows: Macrophage 1 is the VSV-G set in [25]; Macrophage 2 is the CCR5 set in [25]; SupT1 [21]; IMR90 1 is the dividing set in [66]; IMR90 2 is the growth-arrested set in [66]; CD4 T [67]; PBMC [22]; Jurkat 1 is the Mse set in [46]; Jurkat 2 is the Avr set in [46]; Jurkat 3 is the initially bright set in [5]; Jurkat 4 is the initially dark set in [5]; Jurkat p75 knockdown [40] [46]; 293T [40]; 293T Scram [40]; 293T p75 knockdown [40].

We analyzed data from 15 HIV integration site data sets for which we also had transcriptional profiling data on gene activity for that cell type. For each microarray data set, the expression level of *LEDGF/p75*-specific probe sets was ranked relative to all other probe sets on the array for that cell type, thus yielding a value for relative *LEDGF/p75* expression. These values were then plotted against the proportion of HIV integration sites in transcription units for that cell type (Figure 9). This analysis showed that increased relative *LEDGF/p75* mRNA abundance positively correlated with increased HIV integration frequency in transcription units (R^2^ = 0.61; P<0.0001). Figure 9 shows data with experimental LEDGF/p75 knockdowns included (triangles), but the correlation was still significant when the experimental knockdowns were excluded (P<0.0001), indicating that natural variation in LEDGF/p75 levels was functionally significant.

Some of the data in Figure 9 and in previous studies was generated using transformed cell lines, leaving open the question of whether natural variation in LEDGF/p75 levels was functionally important in human primary cells. We repeated the analysis in Figure 9 using only data from human primary cells where LEDGF/p75 levels had not been altered experimentally, and again found a significant positive correlation between integration frequency in genes and *LEDGF/p75* mRNA levels (P = 0.044). These data indicate that natural variation in *LEDGF/p75* expression levels is a significant determinant of integration frequency in transcription units in human primary cells.

## Discussion

Here we report studies of lentiviral integration in two cell types with strong depletions of LEDGF/p75. In the first, we studied the SupT1 human T-cell line with intensified RNAi against LEDGF/p75 described in [37]. Extensive characterization has shown that these cells have stronger knockdowns than those studied previously (e. g. [12], [40]), providing an improved model for the role of LEDGF/p75 in lentiviral integration targeting in human T-cells. In the second cell model, we studied murine cells with a homozygous gene-trap mutation disrupting the *LEDGF/p75* locus [42]. We also presented data on EIAV, extending the collection of lentiviruses shown functionally to be affected by LEDGF/p75. Infectivity for both HIV and EIAV was reduced 5–50 fold in LEDGF/p75-depleted cells, in good agreement with data on HIV and FIV published previously [37], [41]–taken together, these studies firmly establishing that strong LEDGF-p75 knockdowns strongly reduce HIV infectivity. The data reported on target site selection in human cells and murine cells were closely parallel with each other, and also parallel with studies of another murine *LEDGF/p75* mutant [37], [41]. Comparison of integration targeting data reported here to earlier data with weaker knockdowns [40] showed that indeed intensifying the LEDGF/p75 depletion further diminished the proportion of lentiviral integration sites in transcription units. Because more than half of the favoring of transcription units was eliminated by the stronger depletion of LEDGF/p75, we can conclude that the LEDGF/p75-dependent pathway is the predominant pathway for targeting integration to transcription units.

Published studies of integration targeting by LEDGF/p75 have relied on analysis of cells where the LEDGF/p75 levels were artificially reduced—thus there is interest in obtaining data on the effects of LEDGF/p75 in cells naturally expressing different levels of the protein. We took advantage of the observation that different cell types differ reproducibly in their frequency of integration in transcription units [40] to investigate this question. A bioinformatic comparison (Figure 9) showed that higher levels of *LEDGF/p75* expression correlated with higher frequencies of integration in transcription units. The trend achieved significance even when the analysis was restricted to human primary cells only. Thus the study of natural variation in *LEDGF/p75* expression allowed us to extend the idea that LEDGF/p75 directs HIV integration to transcription units in human primary cells without artificially reduced LEDGF/p75 levels.

A simple model holds that LEDGF/p75 directs favored integration into transcription units by tethering. According to this model, one domain of LEDGF/p75 binds to HIV preintegration complexes and the other binds chromatin at active transcription units. Data from artificial tethering studies *in vitro* with fusions of the LEDGF/p75 IBD to a sequence-specific binding domain support this model [56]. The tethering model predicts that LEDGF/p75 should accumulate on active transcription units, but so far this has not been demonstrated experimentally. Similarly, it is not known how LEDGF/p75 recognizes active transcription units. One possible model would be that histone post-translational modifications mark active transcription units and guide LEDGF/p75 binding. Potentially consistent with this idea is the finding that HIV integration is positively correlated with several types of histone post-translational modifications [46].

Curiously, both this study and Shun et al. [41] showed not only a loss of integration targeting in LEDGF/p75-depleted cells, but new favored genomic regions as well. From the previous study alone this might have been an idiosyncrasy of the murine model, but data presented here shows a similar response in human cells. In all LEDGF/p75-depleted cell types in both studies, integration became more favored near transcription start sites and associated CpG islands. The basis for this trend is unknown. It may be that preintegration complexes normally associated with LEDGF/p75 become free to integrate near these sites once LEDGF/p75 was removed. Possibly chromatin at start site regions is particularly accessible and so represents a default target. It is also possible that a more active mechanism is involved. In support of this idea is the finding that MLV integration is strongly favored at start sites [28], [57], while several other integrating elements show near random distributions [22], [55], [58], suggesting that mechanisms exist to guide preferential integration near start sites. A variety of genomic features showed positive correlation with lentiviral integration in both the depleted cells and controls, indicating that cellular systems in addition to LEDGF/p75 also influence integration. As increasingly deep annotation of the human genome accumulates, it may be possible to detect additional associations between lentiviral integration and particular bound proteins, potentially allowing identification of host cell factors operating in the absence of LEDGF/p75.

Finally, data presented here and in [37], [41] emphasizes that LEDGF/p75 is important for efficient HIV replication, suggesting that the interaction between IN and LEDGF/p75 may be a tractable target for antiviral therapy. The structure of a complex of the LEDGF/p75 IBD and the IN catalytic domain have been solved by X-ray crystallography[33], and the interaction surface was found to overlap with the binding site seen previously for the integrase inhibitor tetraphenylarsonium [59]. This supports the idea that small molecule inhibitors, if of high enough affinity, may be able to disrupt binding of LEDGF/p75 to integrase and so abrogate HIV replication.

## Materials and Methods

### Cell lines

MEFs were extracted from wild-type and knockout embryos at 13.5 dpc [60] and cultured in DMEM with 10% FBS, 50 µg/ml gentamycin, 110 µM beta-mercaptoethanol, 1× non-essential amino acids, 100 µM sodium pyruvate. Primary MEFs (prMEFs) were immortalized by the 3T3 protocol, by splitting cells every 3 days to a density of 6×10^4^ cells/ml [61].

TC2 and TL2 are control (“scramble” sequence) and active shRNA-expressing SupT1 cell lines derived in parallel by intensified RNAi. They were established simultaneously from the same parental population, using equivalent MOI transduction with lentiviral vectors that differed only in the 19 nt of the shRNA [37].

### Viral particle production and infections

VSV-G pseudotyped HIV vector particles were produced by Lipofectamine transfection of 293T cells with p156RRLsin-PPTCMVGFPWPRE [62], the packaging construct pCMVdeltaR9 [63], and the vesicular stomatitis virus G-producing pMD.G construct. EIAV vector particles were likewise produced by transfection with p6.1G3CeGFPw (M. Patel and J. Olsen, University of North Carolina, Chapel Hill unpublished), the packaging construct pEV53B [64], and the vesicular stomatitis virus G-producing plasmid pVSVG into 293T cells. Viral supernatant was harvested 38 hours after transfection, filtered through 0.22 µm filters, concentrated by filtration through a Centricon, treated with DNase I, and stored frozen at −80°C. HIV titer was quantified by p24 ELISA.

For EIAV infection of SupT1 cells, cells were plated at 1×10^5^ cells per well of a 24-well plate, infected with between 25–100 µl concentrated DNase I treated virus stock, and all wells were brought to 200 µl final volume with fresh RPMI containing 10% heat-inactivated FBS, 10 units/ml penicillin, 10 µg/ml streptomycin and 50 µg/ml gentamycin (R-10). At 5 hours all well contents were transferred to a 1.5 ml Eppendorf and spun for 10 min at 1000RPMs to pellet cells. Cells were resuspended in 1 ml R-10 and cultured for an additional 76 hrs for integration site cloning or 2 weeks for QPCR analysis. Upon collection, 30–50% of cells expressed GFP as analyzed by fluorescence microscopy.

For HIV infection of MEFs, cells were plated onto 6-well plates at a density of 3×10^5^ cells per well and each well infected with 1 µg p24. For EIAV, cells were plated into 24-well plates at a density of 4×10^4^ cells per well, and each well infected with 100 µl concentrated virus. Infections were performed overnight in the presence of 10 µg/ml DEAE-dextran. 10 independent HIV infections and 5 EIAV infections were performed per genotype. 48 hours after infection, 90% of cells were harvested for integration site cloning and the remainder passaged for an additional 2 weeks to dilute unintegrated products of reverse transcription and used for QPCR analysis of integration efficiency.

### Infectivity tests

For quantitative PCR analysis, infected cells were passaged for 2 weeks following infection to dilute unintegrated products of reverse transcription, then genomic DNA was extracted using the Qiagen DNeasy tissue extraction kit. QPCR using HIV late-RT primers and probe was carried out as described in [44] using 50 ng genomic DNA as template. For EIAV, primer and probe sequences are described in Table S1. 25 ng of SupT1 genomic DNA was used as template, 50 ng of MEF genomic DNA. QPCR was performed using Applied Biosystems 2× FAST universal master mix and Applied Biosystems FAST PCR machine.

For luciferase assays, HIV luciferase reporter virus stock was prepared by transfection of pLai3_envLuc2 [65] and the vesicular stomatitis virus G-producing plasmid pVSVG into 293T cells. Viral supernatant was collected 36 h after transfection, filtered through 0.22-µm filters, concentrated, assayed by p24 and stored frozen at –80°C. For infectivity assay, SupT1 cells plated at 1×10^5^ cells per well of a 24-well plate were infected with various amounts of concentrated DNase treated virus stock. All wells were brought to 1 ml final volume with fresh R-10. Three days later, cells were lysed in 0.5% Triton-X 100 in PBS and luciferase levels were determined using Luciferase Assay System and a Thermo Luminoskan Ascent luminescence counter. All infections were performed in triplicate.

### Integration site cloning

Integration sites were isolated and sequenced by linker-mediated PCR essentially as described previously [46]. Genomic DNA was extracted from infected cells using the Qiagen DNeasy tissue extraction kit. Up to 2 µg of DNA from each infection was digested overnight using MseI. This was followed by digestion to prevent amplification of internal viral fragments (from the 5′ LTR) and plasmid backbone with SacI and DpnI in the case on HIV, and XmaI and DpnI in the case of EIAV. Linkers were then ligated onto digestion products (oligonucleotide sequences can be found in Table S1) and nested PCR performed from ligation products. Nested PCR primers contained 4 or 8 nt barcode sequences between the sequencing primer and LTR-binding portions. These enabled pooling of all PCR products into one sequencing reaction and subsequent separation of sequences by decoding the barcodes. Amplification products were gel-purified and sent to the Interdisciplinary Center for Biotechnology Research at the University of Florida and the Virginia Bioinformatics Institute Core Laboratory Facility for pyrosequencing.

### Bioinformatic analysis

Integration sites were judged to be authentic if the sequences had a best unique hit when aligned to the murine or human genome as appropriate (mm8 and hg18 respectively) using BLAT, and the alignment began within 3bp of the viral LTR end and had >98% sequence identity. Detailed statistical methods are described in [55] and Statistical Reports S1 and S2.

To control for possible biases in isolating integration sites due to restriction enzyme sequence distribution, three-ten matched random controls were computationally generated for each experimental integration site that were the same distance from the closest MseI restriction site as the experimental site.

Integration site counts in various genomic annotations were compared with matched random controls by the Fisher's exact test. Additionally, multiple regression models for integration intensity were applied, as described in [55].

For analysis of correlations with gene activity in murine integration sites (Figure 7 and 8), transcriptional profiling data from wild-type MEFs analyzed on the MGU74Av2 Affymetrix microarray were used. Genes represented on the microarray were ranked by expression level and divided into 4 bins based on expression level. Integration sites found within genes in each bin were counted as a proportion of sites found within genes in all bins. For human expression analysis (Figure 4) data was from [5].

For the analysis of relative gene activity in Figure 9, data from two types Affymetrix chips were used (HU95A and HU133A). Two probe sets querying *LEDGF/p75* but not *p52* were available on each chip (For HU95: 39243_s_at and 37622_r_at; for HU133: 209337_at and 205961_s_at). To account for differences in the sensitivities arising from the different chip designs and probe sets, the values for each cell type were first ranked for each probe set and chip combination, then the ranked values pooled in the final data set.

## Supporting Information

Table S1 Oligonucleotides used in this study
(0.01 MB XLS)

Statistical Report S1 EIAV integration in human cells
(0.35 MB PDF)

Statistical Report S2 EIAV and HIV integration in murine cells
(1.44 MB PDF)
