# Supplementary material for: Role of PSIP1/LEDGF/p75 in Lentiviral Infectivity and Integration Targeting
Source: PLoS One. 2007 Dec 19;2(12):e1340. doi: 10.1371/journal.pone.0001340 (PMC2129110; doi:10.1371/journal.pone.0001340)
Supplement: Statistical Report S1 — EIAV integration in human cells (0.35 MB PDF) [file pone.0001340.s002.pdf]

Association of Genomic Features with Integration

Charles C. Berry

August 30, 2007

Contents

1 Introduction **2**

2 Preference for Genes **4**
2.1 Acembly Genes 4
2.2 refGenes 6
2.3 ensGenes 7
2.4 genScan Genes 9
2.5 uniGenes 11
2.6 oncogenes 14

3 CpG Island Neighborhoods **15**
3.1 1 kilobase neighborhoods 15
3.2 5 kilobase neighborhoods 16
3.3 10 kilobase neighborhoods 16
3.4 25 kilobase neighborhoods 17
3.5 50 kilobase neighborhoods 18

4 Gene Density, Expression 'Density', and CpG Island Density **20**
4.1 25 kilobase Window 20
4.2 50 kilobase Window 24
4.3 100 kilobase Window 29
4.4 250 kilobase Window 34
4.5 500 kilobase Window 39
4.6 1 megabase Window 44
4.7 2 megabase Window 49
4.8 4 megabase Window 54
4.9 8 megabase Window 59
4.10 16 megabase Window 64
4.11 32 megabase Window 69

5 Juxtaposition with Gene Start and End Positions **75**
5.1 Acembly Annotations 75
5.2 RefSeq Annotations 79
5.3 genScan Annotations 83
5.4 uniGene Annotations 87

6 GC content **91**

7 Cytobands **92**

1 Introduction

In this document, I examine the association of integration sites with various genomic features.

The data consist of both actual integration sites and sets of control sites, each set chosen to match the spacing (in bases) from the nearest restriction site (according to the direction in which the sequence was read) to an integration site. The numbers of insertion and matching sites for several data sets are shown below:

```
                  type
Origin.of.data.set insertion match
        a-wt             783  7829
        b-SCRAM          869  8670
        c-LEDGF_KD       157  1550
```

The advantage of choosing 'control' sites that match the spacing from the nearest restriction site is that biases due to location and density of restriction sites are eliminated by applying the classical multinomial logit model (reviewed in [2]). This model allows regression procedures to be applied to the study of integration intensity as a function of genomic features. The `clogit` function of the R `survival` library) implements estimation and fitting for such models along with the usual likelihood ratio and Wald tests.

The distribution of relative frequency of insertions across the chromosomes is given in this barplot:

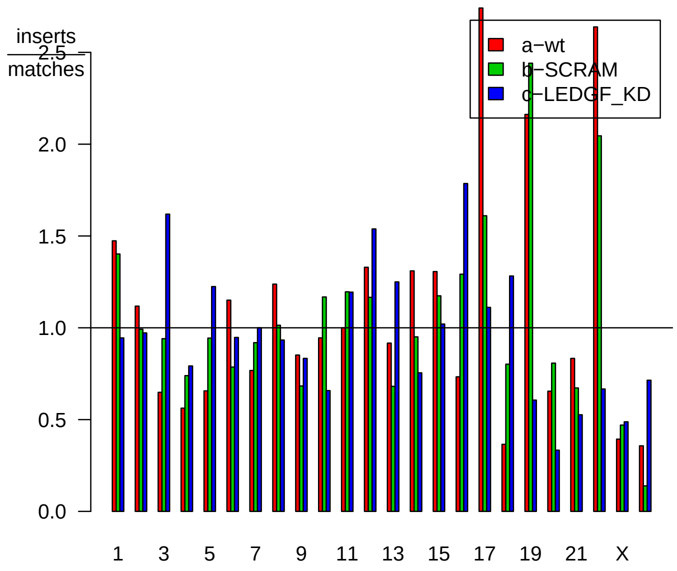

It seems evident that there are some chromosomes that are particularly favored for integration. This is reinforced by a test of statistical significance. The test performed used the likelihood ratio statistic for the multinomial logit model (reviewed in [2]) as implemented by the `clogit` function of the R `survival` library). The null hypothesis tested is that the ratio of true integration events to matched control sites is constant across all chromosomes. This test attains a p-value of $< 2.22e - 16$.

2 Preference for Genes

2.1 Acembly Genes

Here we examine the preference that integration events have for genes. In the following plot we show the relative frequency of integrations in genes according to the 'Acembly' annotation. The bars grouped over the label "In Gene" give the relative frequency of integration events (compared to control sites) between bases located within Acembly gene annotations, while the label "Not in Gene" give the relative frequency of integration events (compared to control sites) between bases not located within Acembly gene annotations.

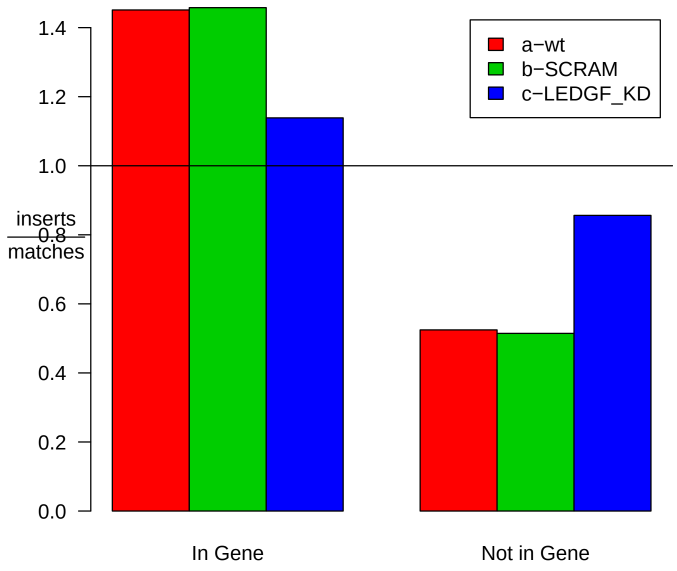

It seems evident that there is a strong tendency for insertions to occur in genes. A formal test of significance bears this out with a p-value of $< 2.22e-16$. Also, it appears that the tendency of different viruses to integrate into genes varies, and a test for this hypothesis attains 0.00042905. Here is the table of coefficients of the log ratio of intensities for true insertion sites versus control insertion sites along with their standard errors, z statistics, and p-values for each data set:

	coef	se	z	p
a-wt	1.01	0.0849	11.90	1.07e-32
b-SCRAM	1.04	0.0814	12.80	1.17e-37
c-LEDGF_KD	0.31	0.1690	1.83	6.71e-02

As is evident, there are some differences in the coefficients. The largest coefficient is seen in the b-SCRAM data set, while the smallest is seen in the c-LEDGF$_K Ddataset.$

In the following plot we show the relative frequency of insertions in exons according to the 'Acembly' annotation. The bars grouped over the label "In Exon" give the relative frequency of integration events (compared to control sites) between bases located in exons according to the Acembly annotation, while the label "Not in Exon" give the relative frequency of integration events (compared to control sites) between bases not located in exons according to the Acembly gene annotation.

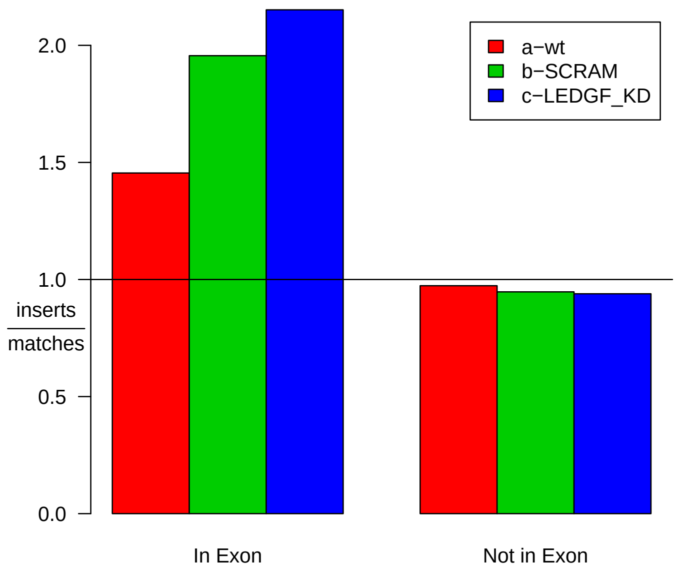

Here is the table of coefficients of the log ratio of intensities for true insertion sites versus control insertion sites along with their standard errors, z statistics, and p-values for each data set:

	coef	se	z	p
a-wt	1.28e-05	0.143	8.92e-05	1.00000
b-SCRAM	3.31e-01	0.124	2.66e+00	0.00773
c-LEDGF_KD	7.27e-01	0.291	2.49e+00	0.01270

The model on which these coefficients are based include terms for whether the site is in a gene or not. Thus, the effect shown as "In Exon" is net of that due to being in a gene. Note that in the barplot above the 'Not in Exon' bars include both the introns and intergenic regions, so the impression given by the

table may differ from that for the barplot.

2.2 refGenes

Here we examine the preference that insertions have for genes. In the following plot we show the relative frequency of insertions in genes according to the 'refGene' annotation.

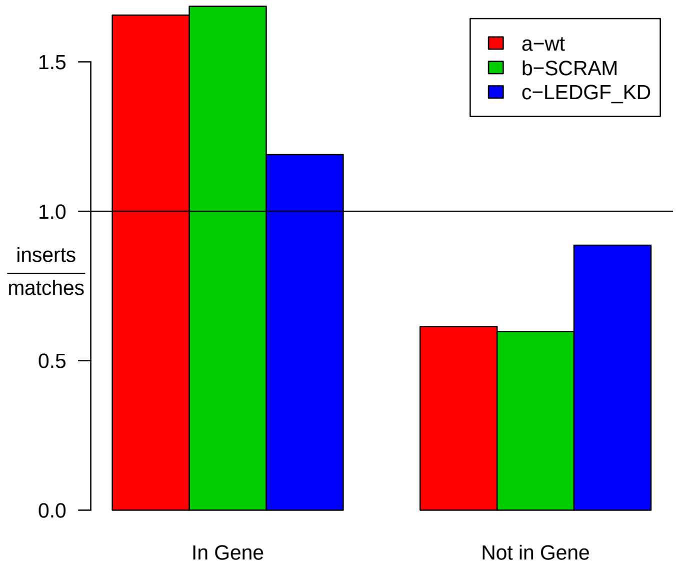

It seems evident that there is a strong tendency for insertions to occur in genes. A formal test of significance bears this out with a p-value of $< 2.22e-16$. Also, it appears that the tendency of different viruses to integrate into genes varies, and a test for this hypothesis attains 0.0003602. Here is the table of coefficients of the log ratio of intensities for true insertion sites versus control insertion sites along with their standard errors, z statistics, and p-values for each data set:

	coef	se	z	p
a-wt	0.998	0.0775	12.90	5.62e-38
b-SCRAM	1.030	0.0734	14.10	6.09e-45
c-LEDGF_KD	0.314	0.1690	1.86	6.30e-02

As is evident, there are some differences in the coefficients. The largest coefficient is seen in the b-SCRAM data set, while the smallest is seen in the c-LEDGF$_K Ddataset$.

In the following plot we show the relative frequency of insertions in exons according to the 'refGene' annotation.

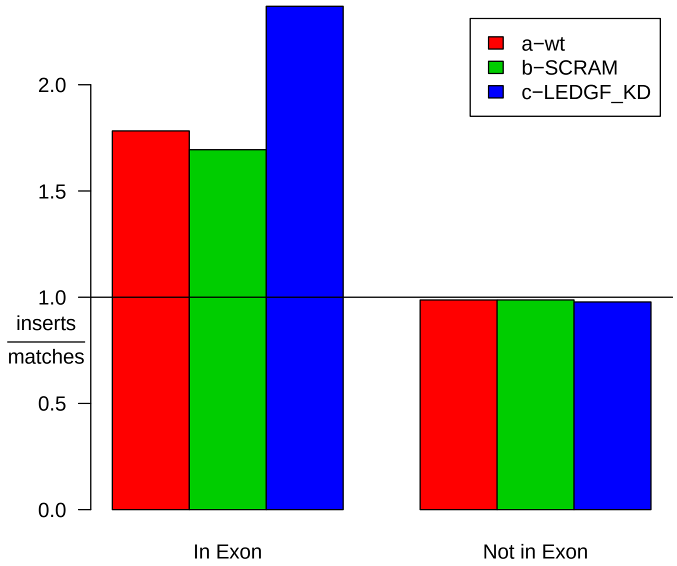

Here is the table of coefficients of the log ratio of intensities for true insertion sites versus control insertion sites along with their standard errors, z statistics, and p-values for each data set:

	coef	se	z	p
a-wt	0.0771	0.232	0.332	0.740
b-SCRAM	0.0228	0.213	0.107	0.915
c-LEDGF_KD	0.7360	0.475	1.550	0.121

The model on which these coefficients are based include terms for whether the site is in a gene or not. Thus, the effect shown as "In Exon" is net of that due to being in a gene.

2.3 ensGenes

Here we examine the preference that insertions have for genes. In the following plot we show the relative frequency of insertions in genes according to the 'ensGene' annotation.

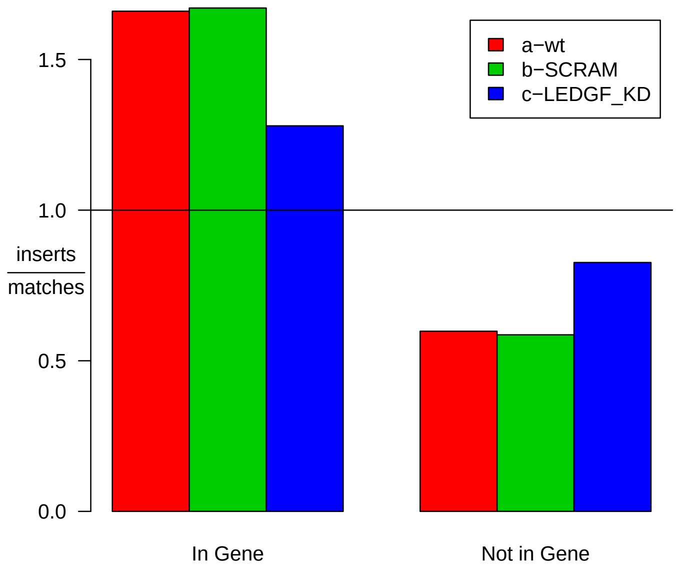

It seems evident that there is a strong tendency for insertions to occur in genes. A formal test of significance bears this out with a p-value of $< 2.22e-16$. Also, it appears that the tendency of different viruses to integrate into genes varies, and a test for this hypothesis attains 0.0051371. Here is the table of coefficients of the log ratio of intensities for true insertion sites versus control insertion sites along with their standard errors, z statistics, and p-values for each data set:

	coef	se	z	p
a-wt	1.020	0.0776	13.10	1.90e-39
b-SCRAM	1.040	0.0739	14.10	2.99e-45
c-LEDGF_KD	0.458	0.1680	2.72	6.47e-03

As is evident, there are some differences in the coefficients. The largest coefficient is seen in the b-SCRAM data set, while the smallest is seen in the c-LEDGF$_K Ddataset$.

In the following plot we show the relative frequency of insertions in exons according to the 'ensGene' annotation.

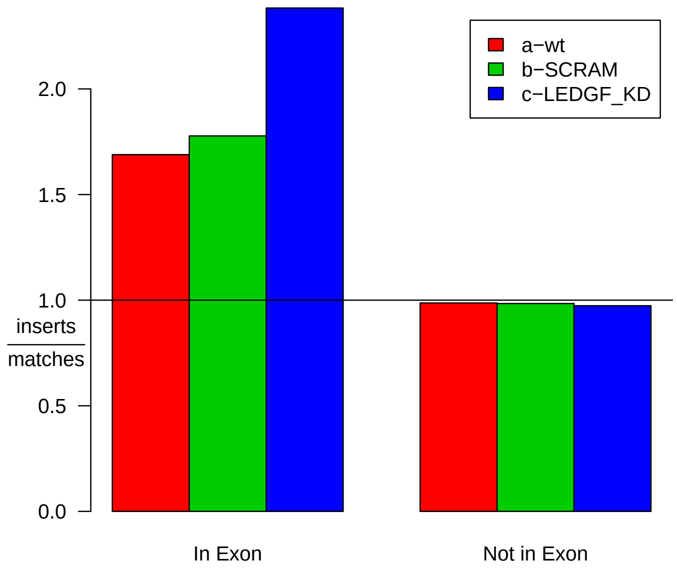

Here is the table of coefficients of the log ratio of intensities for true insertion sites versus control insertion sites along with their standard errors, z statistics, and p-values for each data set:

	coef	se	z	p
a-wt	0.0118	0.222	0.0532	0.958
b-SCRAM	0.0818	0.200	0.4090	0.682
c-LEDGF_KD	0.6850	0.441	1.5500	0.121

The model on which these coefficients are based include terms for whether the site is in a gene or not. Thus, the effect shown as ”In Exon” is net of that due to being in a gene.

2.4 genScan Genes

Here we examine the preference that insertions have for genes. In the following plot we show the relative frequency of insertions in genes according to the ’genScan’ annotation.

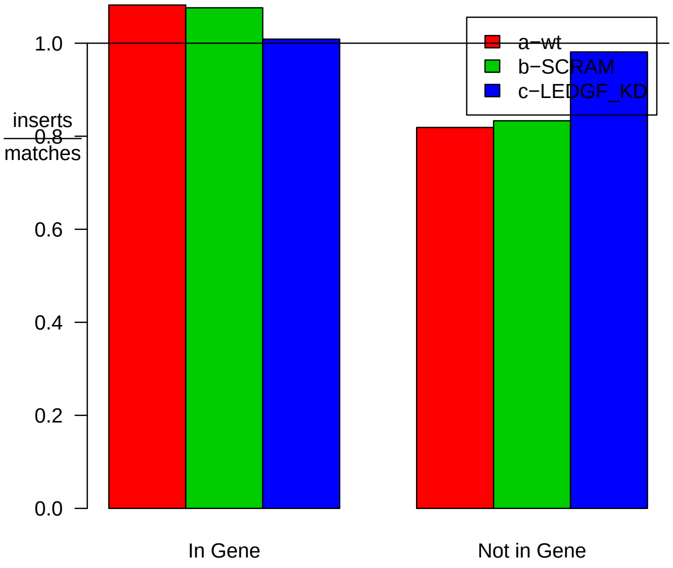

It seems evident that there is a strong tendency for insertions to occur in genes. A formal test of significance bears this out with a p-value of $8.781e-06$. Also, it appears that the tendency of different viruses to integrate into genes varies, and a test for this hypothesis attains 0.39564. Here is the table of coefficients of the log ratio of intensities for true insertion sites versus control insertion sites along with their standard errors, z statistics, and p-values for each data set:

	coef	se	z	p
a-wt	0.2800	0.0858	3.2700	0.00108
b-SCRAM	0.2570	0.0805	3.1900	0.00141
c-LEDGF_KD	0.0089	0.1810	0.0492	0.96100

As is evident, there are some differences in the coefficients. The largest coefficient is seen in the a-wt data set, while the smallest is seen in the c-LEDGF$_{K}Ddataset$.

In the following plot we show the relative frequency of insertions in exons according to the 'genScan' annotation.

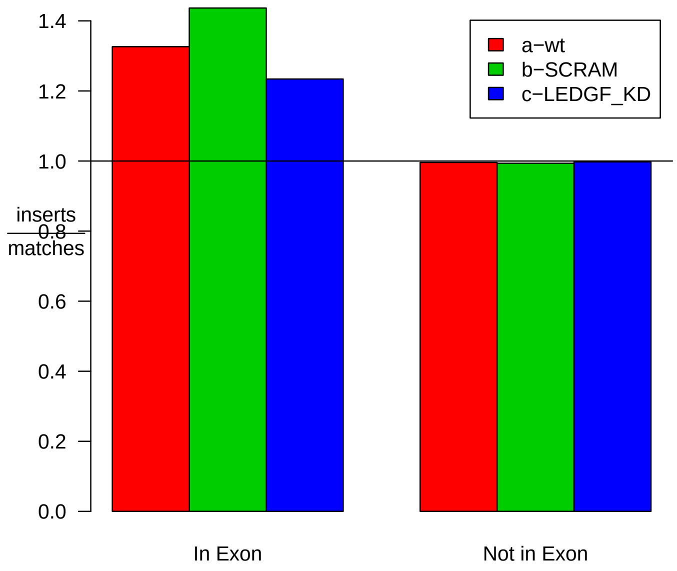

Here is the table of coefficients of the log ratio of intensities for true insertion sites versus control insertion sites along with their standard errors, z statistics, and p-values for each data set:

	coef	se	z	p
a-wt	0.209	0.297	0.705	0.481
b-SCRAM	0.304	0.256	1.190	0.234
c-LEDGF_KD	0.227	0.763	0.298	0.766

The model on which these coefficients are based include terms for whether the site is in a gene or not. Thus, the effect shown as ”In Exon” is net of that due to being in a gene.

2.5 uniGenes

Here we examine the preference that insertions have for genes. In the following plot we show the relative frequency of insertions in genes according to the ’uniGene’ annotation.

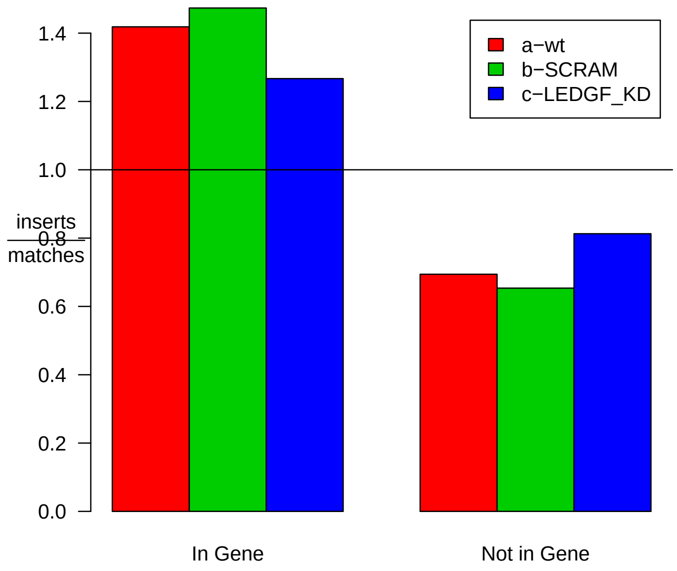

It seems evident that there is a strong tendency for insertions to occur in genes. A formal test of significance bears this out with a p-value of $< 2.22e - 16$. Also, it appears that the tendency of different viruses to integrate into genes varies, and a test for this hypothesis attains 0.11295. Here is the table of coefficients of the log ratio of intensities for true insertion sites versus control insertion sites along with their standard errors, z statistics, and p-values for each data set:

	coef	se	z	p
a-wt	0.713	0.0764	9.33	1.07e-20
b-SCRAM	0.814	0.0733	11.10	1.06e-28
c-LEDGF_KD	0.439	0.1680	2.62	8.86e-03

As is evident, there are some differences in the coefficients. The largest coefficient is seen in the b-SCRAM data set, while the smallest is seen in the c-LEDGF$_{K}Ddataset.$

In the following plot we show the relative frequency of insertions in exons according to the 'uniGene' annotation.

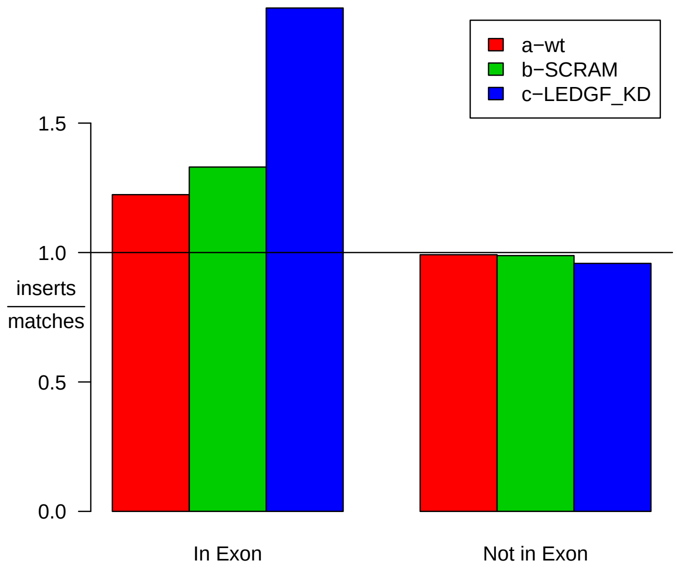

Here is the table of coefficients of the log ratio of intensities for true insertion sites versus control insertion sites along with their standard errors, z statistics, and p-values for each data set:

	coef	se	z	p
a-wt	-0.169	0.187	-0.903	0.366
b-SCRAM	-0.106	0.176	-0.603	0.547
c-LEDGF_KD	0.401	0.334	1.200	0.230

The model on which these coefficients are based include terms for whether the site is in a gene or not. Thus, the effect shown as ”In Exon” is net of that due to being in a gene.

2.6 oncogenes

Here we examine the preference that insertions have for oncogenes. In the following plot we show the relative frequency of insertions with 50kb of an oncogene 5' end.

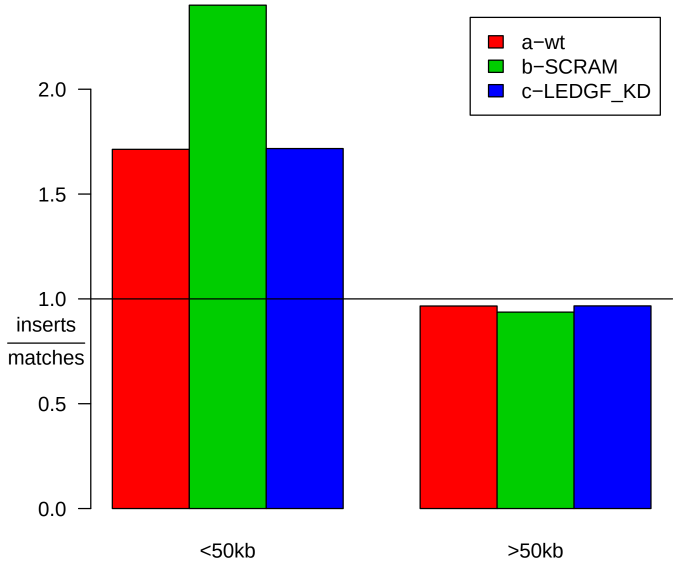

A formal test of oncogenic insertion returns p-value of $2.9495e-15$. The tendency of different viruses to integrate near oncogenes may vary, and a test for this hypothesis attains 0.12711. Here is the table of coefficients of the log ratio of intensities for true insertion sites versus control insertion sites along with their standard errors, z statistics, and p-values for each data set:

	coef	se	z	p
a-wt	-0.574	0.144	-3.98	6.81e-05
b-SCRAM	-0.942	0.123	-7.64	2.17e-14
c-LEDGF_KD	-0.591	0.326	-1.81	6.99e-02
a-wt	NA	0.000	NA	NA
b-SCRAM	NA	0.000	NA	NA
c-LEDGF_KD	NA	0.000	NA	NA

As is evident, there are some differences in the coefficients. The largest coefficient is seen in the a-wt data set, while the smallest is seen in the b-SCRAM data set.

3 CpG Island Neighborhoods

Here we study the effect of being in the neighborhood of CpG Islands. Following Wu et al [3], who found that the neighborhoods within ±1kb of CpG islands are enriched for MLV insertions, we study such neighborhoods.

3.1 1 kilobase neighborhoods

The following plot shows the effect of being in or within ±1kb of a CpG island:

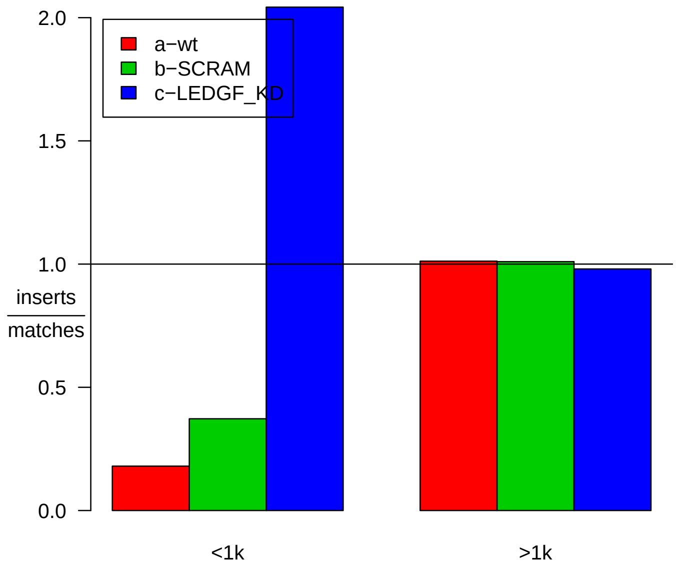

A formal test of significance comparing the difference attains a p-value of 0.00054143. A test for differences between viruses attains 0.058116. Here is the table of coefficients of the log ratio of intensities for true insertion sites versus control insertion sites along with their standard errors, z statistics, and p-values for each data set:

	coef	se	z	p
a-wt	-1.730	0.715	-2.42	0.0154
b-SCRAM	-0.993	0.456	-2.17	0.0297
c-LEDGF_KD	0.331	0.542	0.61	0.5420

The largest coefficient is seen in the c-LEDGF$_K Ddataset, whilethesmallestisseeninthea-wtdataset.$

3.2 5 kilobase neighborhoods

The following plot shows the effect of being in or within ±5kb of a CpG island:

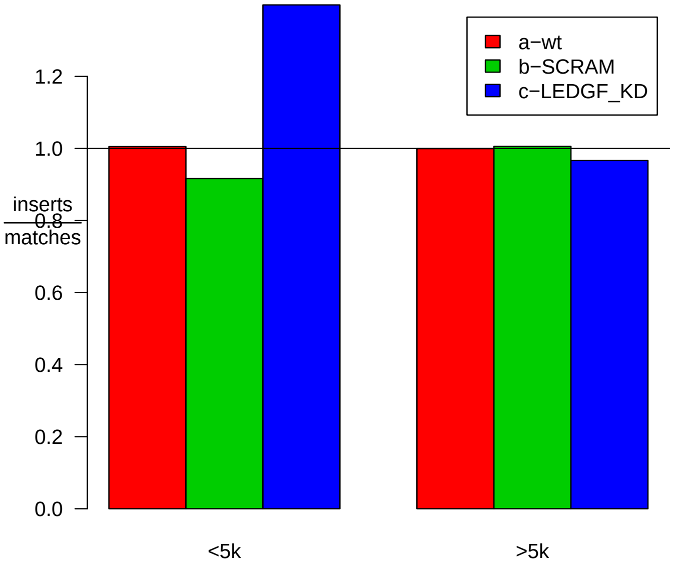

A formal test of significance comparing the difference attains a p-value of 0.9014. A test for differences between viruses attains 0.59393. Here is the table of coefficients of the log ratio of intensities for true insertion sites versus control insertion sites along with their standard errors, z statistics, and p-values for each data set:

	coef	se	z	p
a-wt	0.00581	0.146	0.0398	0.968
b-SCRAM	-0.09100	0.148	-0.6130	0.540
c-LEDGF_KD	0.24000	0.285	0.8410	0.400

The largest coefficient is seen in the c-LEDGF$_KDdataset, whilethesmallestisseenintheb-SCRAMdataset.$

3.3 10 kilobase neighborhoods

The following plot shows the effect of being in or within ±10kb of a CpG island:

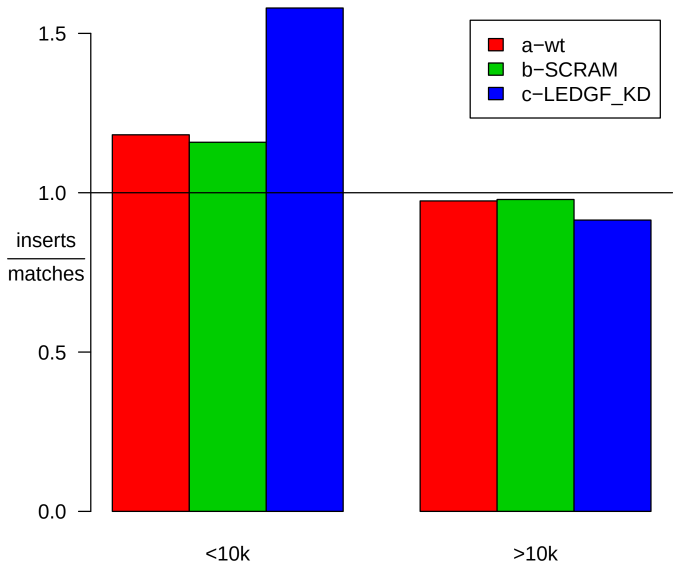

A formal test of significance comparing the difference attains a p-value of 0.0031006. A test for differences between viruses attains 0.45534. Here is the table of coefficients of the log ratio of intensities for true insertion sites versus control insertion sites along with their standard errors, z statistics, and p-values for each data set:

	coef	se	z	p
a-wt	0.196	0.107	1.82	0.0681
b-SCRAM	0.173	0.105	1.65	0.0990
c-LEDGF_KD	0.472	0.214	2.20	0.0277

The largest coefficient is seen in the c-LEDGF$_K Ddataset, whilethesmallestisseenintheb-$ $SCRAMdataset.$

3.4 25 kilobase neighborhoods

The following plot shows the effect of being in or within ±25kb of a CpG island:

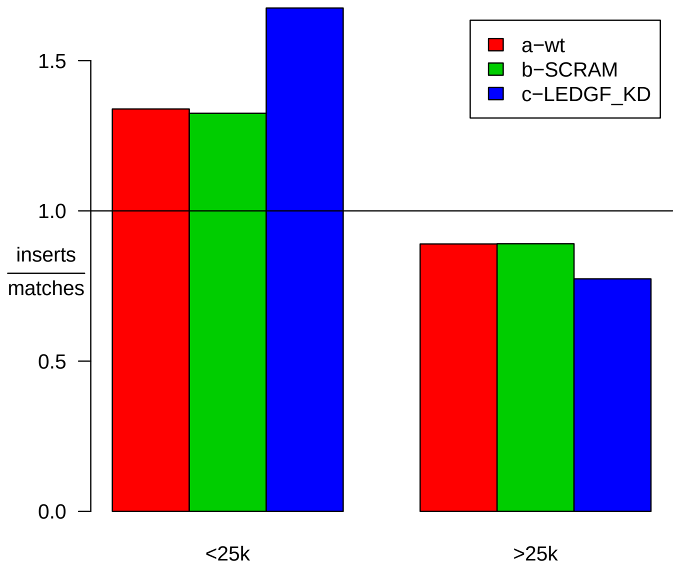

A formal test of significance comparing the difference attains a p-value of $5.2149e-16$. A test for differences between viruses attains 0.19288. Here is the table of coefficients of the log ratio of intensities for true insertion sites versus control insertion sites along with their standard errors, z statistics, and p-values for each data set:

	coef	se	z	p
a-wt	0.413	0.0809	5.10	3.38e-07
b-SCRAM	0.401	0.0761	5.26	1.41e-07
c-LEDGF_KD	0.738	0.1730	4.26	2.01e-05

The largest coefficient is seen in the c-LEDGF$_K Ddataset, whilethesmallestisseenintheb-SCRAMdataset.$

3.5 50 kilobase neighborhoods

The following plot shows the effect of being in or within $\pm$50kb of a CpG island:

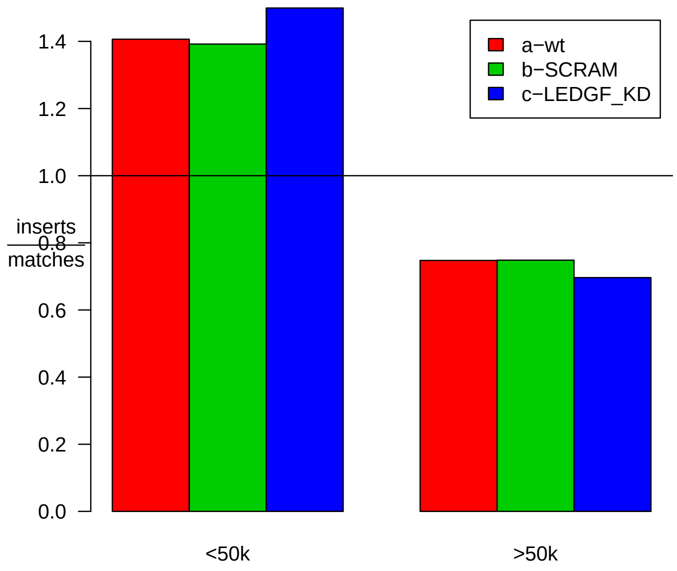

A formal test of significance comparing the difference attains a p-value of $< 2.22e - 16$. A test for differences between viruses attains 0.8211. Here is the table of coefficients of the log ratio of intensities for true insertion sites versus control insertion sites along with their standard errors, z statistics, and p-values for each data set:

	coef	se	z	p
a-wt	0.639	0.0759	8.42	3.68e-17
b-SCRAM	0.629	0.0719	8.75	2.21e-18
c-LEDGF_KD	0.744	0.1700	4.37	1.22e-05

The largest coefficient is seen in the c-LEDGF$_K Ddataset, whilethesmallestisseenintheb- SCRAMdataset.$

4 Gene Density, Expression 'Density', and CpG Island Density

In this section the association with gene density is examined. For expression analysis, the 'genes' that are counted are the genes represented on the microarray. In addition, we the number of such genes expressed at various levels. The levels are

low.ex Count genes whose expression is in the upper half and divide by number of bases

med.ex Count genes whose expression is in the upper $1/8^{th}$ and divide by number of bases

high.ex Count genes whose expression is in the upper $1/16^{th}$ and divide by number of bases

The bolded terms are used as abbreviations in what follows. The abbreviation **dens** is used to indicate gene density as number of genes per base.

4.1 25 kilobase Window

In the barplot that follows we examine the association of insertion sites with gene density in a 25 kilobase window surrounding each locus. More such plots will follow and the method of their construction is always to try to divide the data according to the deciles of density. However, it often happens that there is a very skewed distribution of density and even the 90th percentile is zero. In that case, the barplots simply show the sites for which the density is zero and those for which it is non-zero. If there are fewer than ten groups of bars, the groupings contain ten percent of the sites each except for the leftmost grouping which will contain all of the remaining sites.

Also note that the title of the plot contains clues as to its content; the prefix indicates the type of variable studied while the suffix indicates the window width in the number of bases. The p-value given is the result of fitting a cubic polynomial to the gene density values.

The following expression data and probe set were used for this report:

```
[1] "SupT1-HU95"
```

```
[1] "HG-U95"
```

```
Density data too sparse for barplot
```

```
             coef    se    z        p
a-wt        0.651 0.112 5.80 6.52e-09
b-SCRAM     0.533 0.113 4.70 2.64e-06
c-LEDGF_KD  0.369 0.275 1.34 1.80e-01
```

Here are the results for expression density. First, we count just genes that are in the upper half.

```
Density data too sparse for barplot

             coef    se    z        p
a-wt        0.859 0.140 6.16 7.46e-10
b-SCRAM     0.618 0.145 4.26 2.02e-05
c-LEDGF_KD  0.791 0.331 2.39 1.67e-02
```

Now we count genes in the upper $1/8^{th}$:

```
Density data too sparse for barplot

            coef    se    z         p
a-wt       0.768 0.198 3.87 1.09e-04
b-SCRAM    0.896 0.187 4.80 1.60e-06
c-LEDGF_KD 0.910 0.467 1.95 5.14e-02
```

And here we count genes in the upper $1/16^{th}$:

```
Density data too sparse for barplot

             coef    se    z        p
a-wt        0.421 0.342 1.23 0.218000
b-SCRAM     0.912 0.251 3.63 0.000284
c-LEDGF_KD  1.430 0.532 2.68 0.007340
```

Here the effect of density of CpG islands is studied:

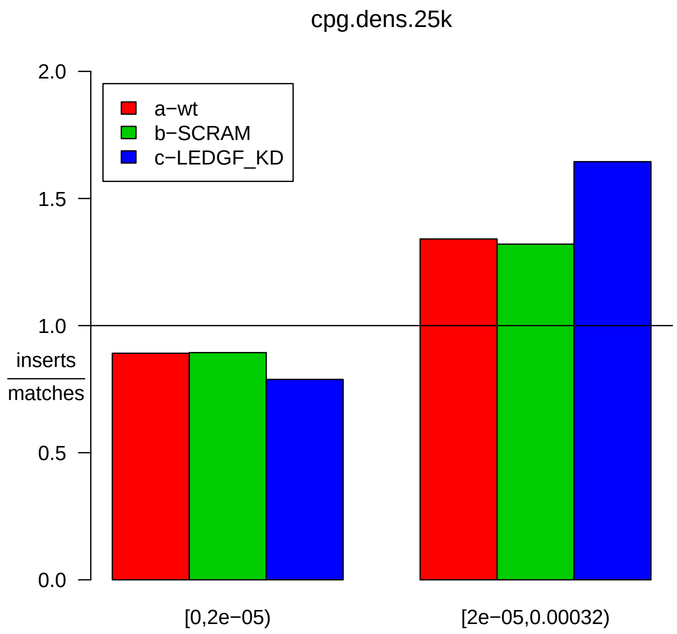

	coef	se	z	p
a-wt	0.413	0.0812	5.08	3.71e-07
b-SCRAM	0.389	0.0764	5.10	3.43e-07
c-LEDGF_KD	0.732	0.1740	4.22	2.47e-05

4.2 50 kilobase Window

In the barplot that follows we examine the association of insertion sites with expression density in a 50 kilobase window surrounding each locus. First, we count just the number of genes represented on the chip.

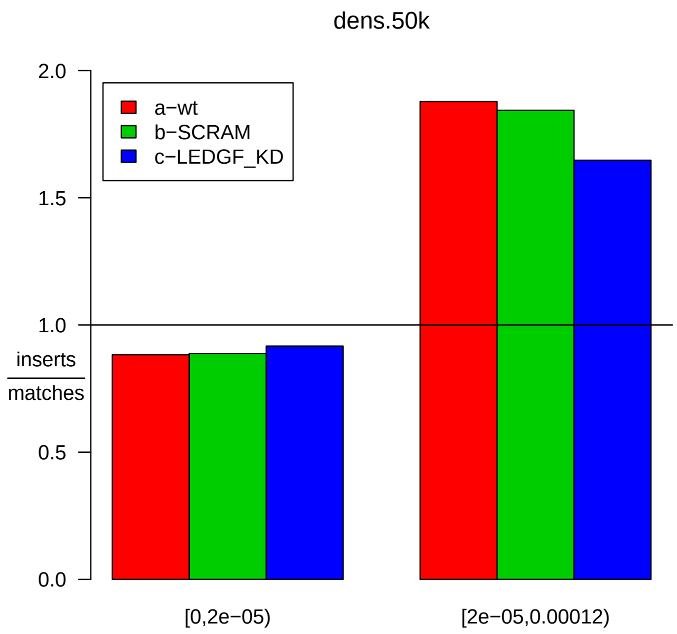

	coef	se	z	p
a-wt	0.716	0.0891	8.04	8.94e-16
b-SCRAM	0.742	0.0853	8.70	3.30e-18
c-LEDGF_KD	0.516	0.2140	2.41	1.59e-02

Here are the results for expression density. First, we count just genes that are in the upper half.

```
Density data too sparse for barplot
```

	coef	se	z	p
a-wt	0.907	0.107	8.49	2.04e-17
b-SCRAM	0.819	0.103	7.99	1.33e-15
c-LEDGF_KD	0.971	0.244	3.98	7.01e-05

Now we count genes in the upper $1/8^{th}$:

```
Density data too sparse for barplot

              coef    se    z         p
a-wt         0.795 0.150 5.28 1.28e-07
b-SCRAM      0.947 0.134 7.06 1.66e-12
c-LEDGF_KD   1.160 0.313 3.70 2.18e-04
```

And here we count genes in the upper $1/16^{th}$:

```
Density data too sparse for barplot

            coef    se    z        p
a-wt       0.635 0.231 2.75 5.96e-03
b-SCRAM    0.921 0.177 5.19 2.06e-07
c-LEDGF_KD 1.460 0.383 3.81 1.41e-04
```

Here the effect of density of CpG islands is studied:

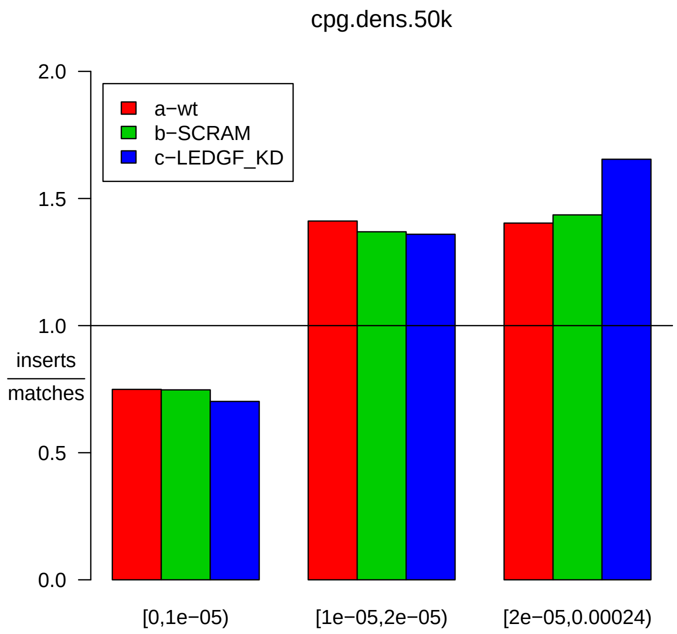


```
             coef     se    z         p
a-wt        0.637 0.0758 8.40 4.51e-17
b-SCRAM     0.629 0.0719 8.75 2.08e-18
c-LEDGF_KD  0.758 0.1700 4.45 8.44e-06
```

4.3 100 kilobase Window

In the barplot that follows we examine the association of insertion sites with expression density in a 100 kilobase window surrounding each locus. First, we count just the number of genes represented on the chip.

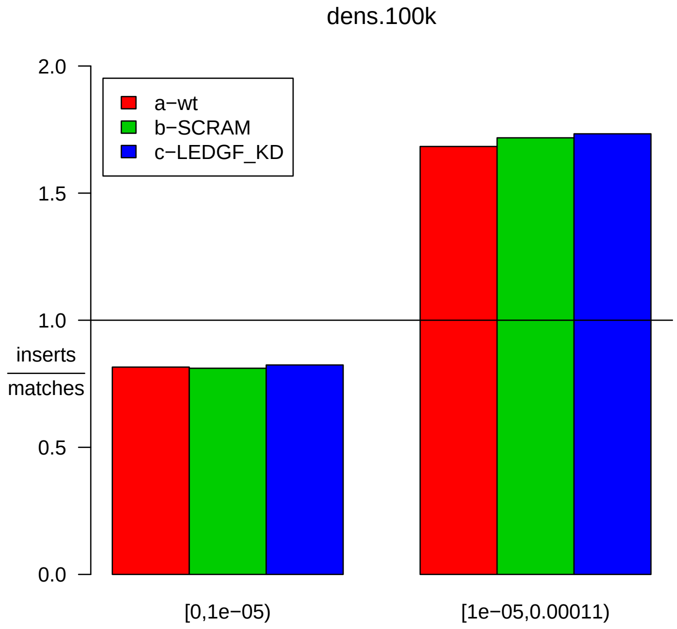

	coef	se	z	p
a-wt	0.735	0.0781	9.42	4.62e-21
b-SCRAM	0.815	0.0745	10.90	7.57e-28
c-LEDGF_KD	0.683	0.1830	3.74	1.81e-04

Here are the results for expression density. First, we count just genes that are in the upper half.

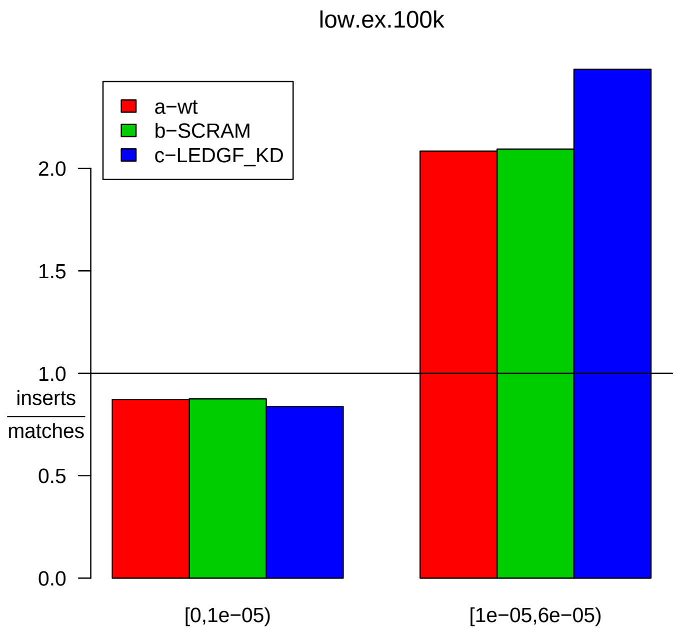

	coef	se	z	p
a-wt	0.887	0.0866	10.20	1.28e-24
b-SCRAM	0.915	0.0834	11.00	4.89e-28
c-LEDGF_KD	1.090	0.2010	5.42	5.94e-08

Now we count genes in the upper $1/8^{th}$:

```
Density data too sparse for barplot

             coef    se    z         p
a-wt        0.865 0.112 7.74 9.85e-15
b-SCRAM     0.956 0.104 9.20 3.70e-20
c-LEDGF_KD 1.150 0.245 4.71 2.46e-06
```

And here we count genes in the upper $1/16^{th}$:

```
Density data too sparse for barplot

            coef    se    z         p
a-wt       0.783 0.155 5.07 4.07e-07
b-SCRAM    0.797 0.136 5.85 4.80e-09
c-LEDGF_KD 1.110 0.315 3.51 4.48e-04
```

Here the effect of density of CpG islands is studied:

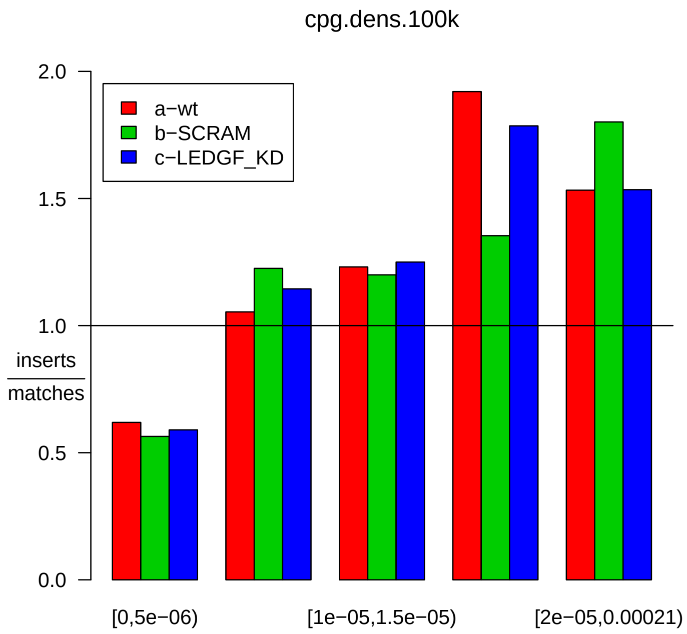

	coef	se	z	p
a-wt	0.695	0.0759	9.16	5.00e-20
b-SCRAM	0.644	0.0720	8.94	3.82e-19
c-LEDGF_KD	0.667	0.1710	3.91	9.22e-05

4.4 250 kilobase Window

In the barplot that follows we examine the association of insertion sites with expression density in a 250 kilobase window surrounding each locus. First, we count just the number of genes represented on the chip.

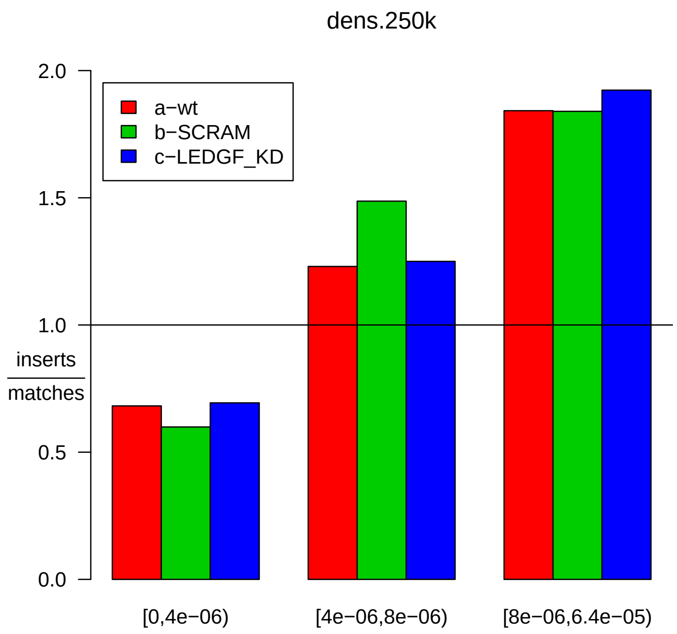

	coef	se	z	p
a-wt	0.791	0.0771	10.30	1.01e-24
b-SCRAM	0.998	0.0751	13.30	2.38e-40
c-LEDGF_KD	0.836	0.1740	4.82	1.47e-06

Here are the results for expression density. First, we count just genes that are in the upper half.

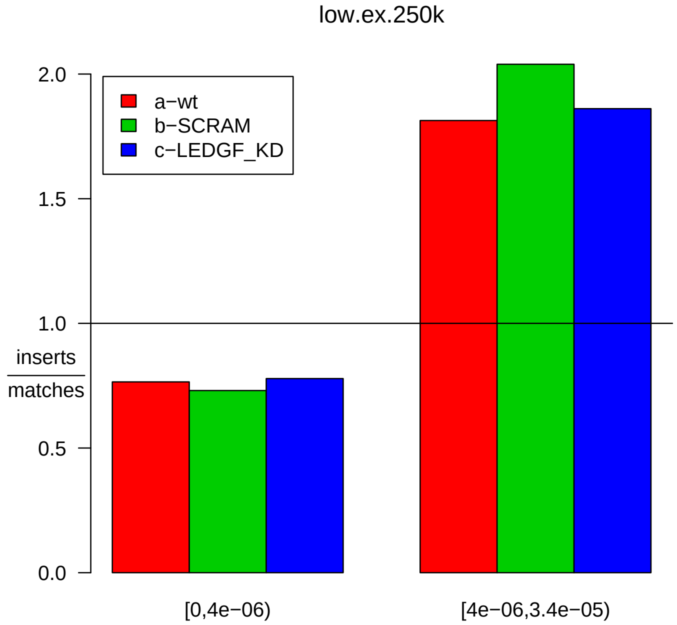

	coef	se	z	p
a-wt	0.907	0.0759	12.00	6.18e-33
b-SCRAM	1.030	0.0726	14.20	4.79e-46
c-LEDGF_KD	0.858	0.1750	4.91	9.02e-07

Now we count genes in the upper $1/8^{th}$:

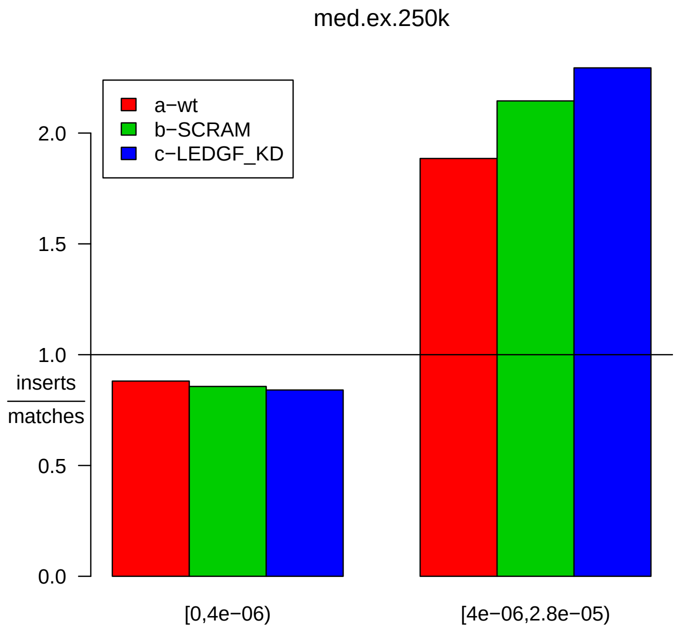

	coef	se	z	p
a-wt	0.897	0.0856	10.50	9.92e-26
b-SCRAM	1.010	0.0813	12.50	1.36e-35
c-LEDGF_KD	0.946	0.1950	4.85	1.24e-06

And here we count genes in the upper $1/16^{th}$:

```
Density data too sparse for barplot

             coef    se    z         p
a-wt        1.020 0.102 9.92 3.31e-23
b-SCRAM     0.848 0.100 8.48 2.16e-17
c-LEDGF_KD  0.888 0.240 3.70 2.18e-04
```

Here the effect of density of CpG islands is studied:

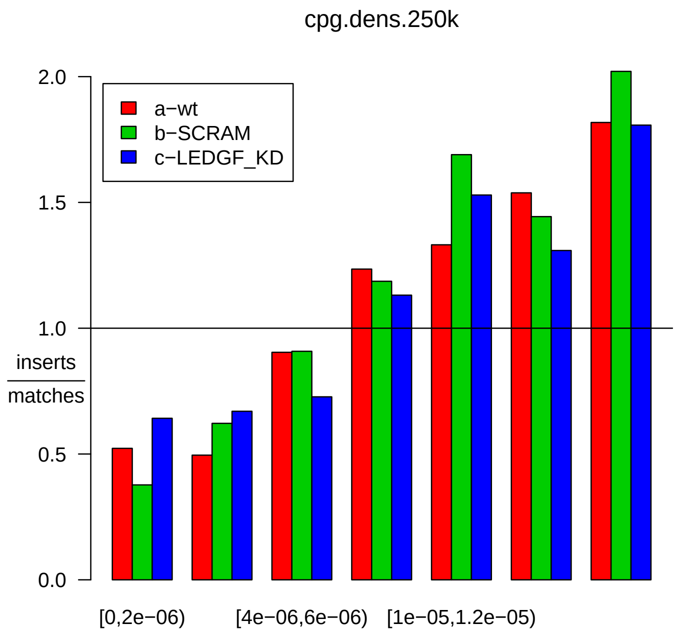


```
            coef     se     z        p
a-wt       0.897 0.0802 11.20 4.83e-29
b-SCRAM    0.978 0.0771 12.70 6.51e-37
c-LEDGF_KD 0.725 0.1750  4.13 3.56e-05
```

4.5 500 kilobase Window

In the barplot that follows we examine the association of insertion sites with expression density in a 500 kilobase window surrounding each locus. First, we count just the number of genes represented on the chip.

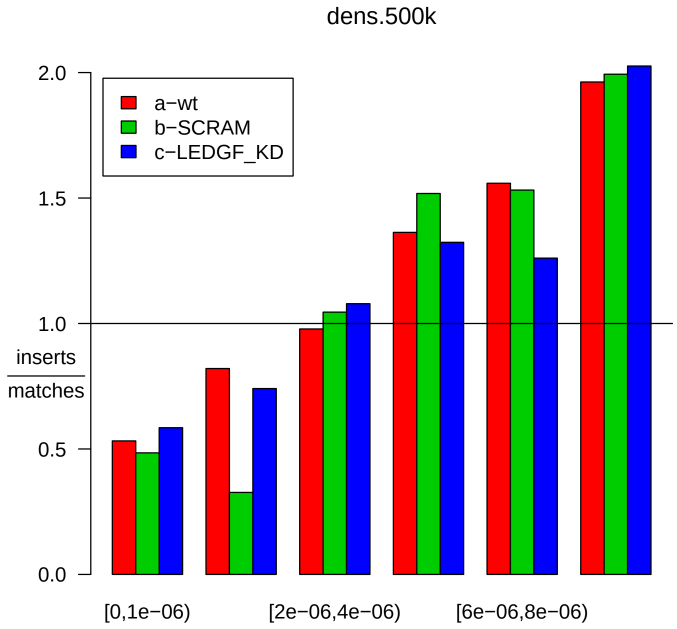

	coef	se	z	p
a-wt	0.862	0.0756	11.40	4.11e-30
b-SCRAM	0.947	0.0725	13.10	4.82e-39
c-LEDGF_KD	0.775	0.1720	4.51	6.56e-06

Here are the results for expression density. First, we count just genes that are in the upper half.

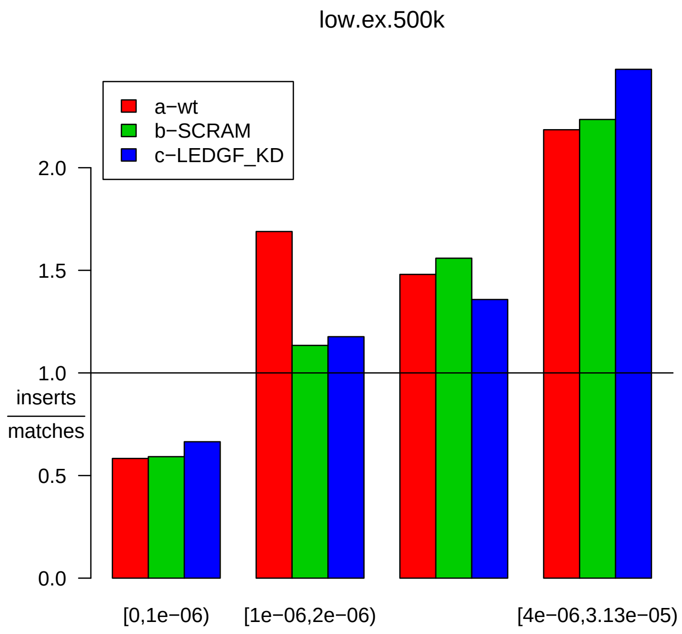

	coef	se	z	p
a-wt	1.080	0.0789	13.70	1.02e-42
b-SCRAM	1.030	0.0743	13.90	1.02e-43
c-LEDGF_KD	0.821	0.1730	4.75	2.00e-06

Now we count genes in the upper $1/8^{th}$:

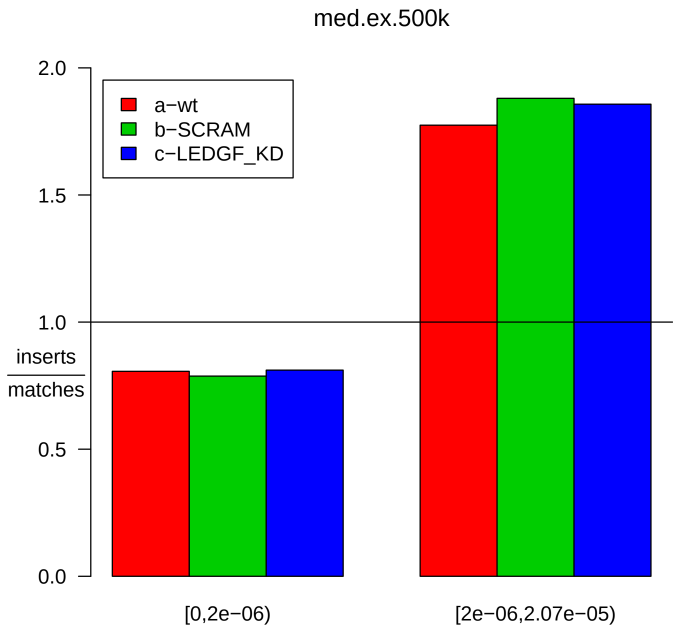

	coef	se	z	p
a-wt	0.873	0.0773	11.30	1.57e-29
b-SCRAM	0.930	0.0733	12.70	7.46e-37
c-LEDGF_KD	0.817	0.1770	4.61	4.05e-06

And here we count genes in the upper $1/16^{th}$:

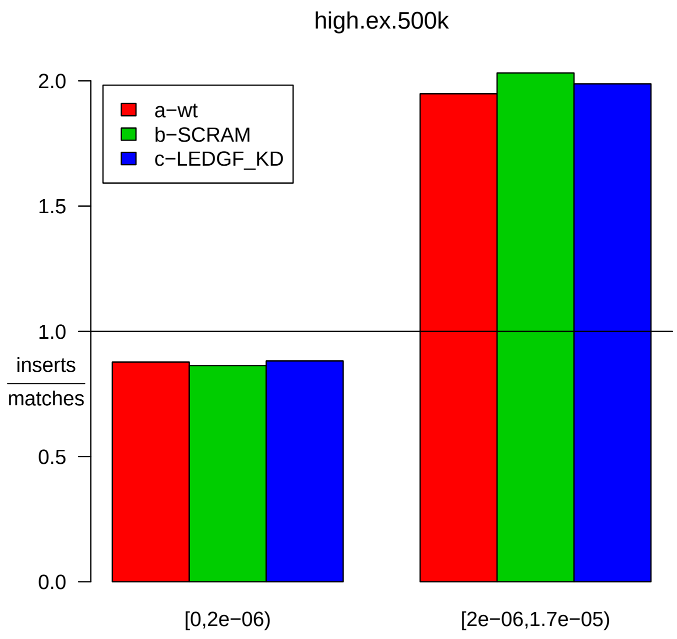

	coef	se	z	p
a-wt	0.988	0.0850	11.60	3.32e-31
b-SCRAM	0.890	0.0819	10.90	1.57e-27
c-LEDGF_KD	0.805	0.2000	4.03	5.57e-05

Here the effect of density of CpG islands is studied:

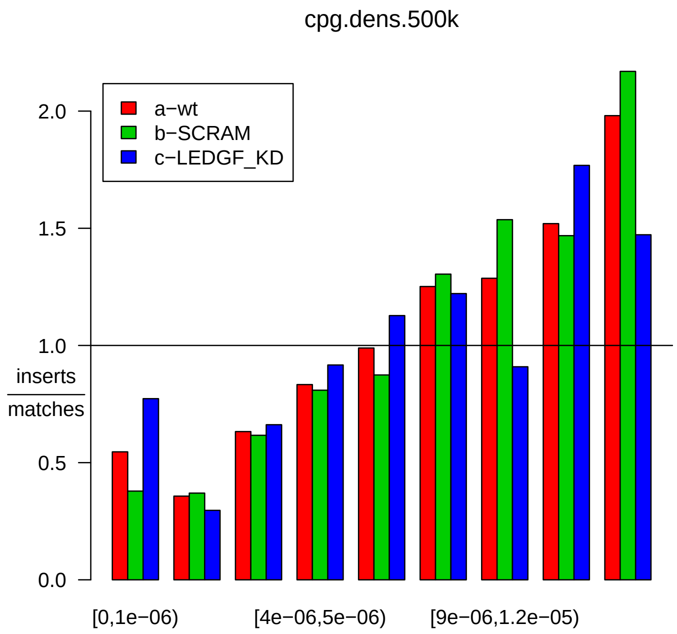

	coef	se	z	p
a-wt	0.889	0.0795	11.20	5.18e-29
b-SCRAM	1.010	0.0773	13.00	1.09e-38
c-LEDGF_KD	0.596	0.1720	3.46	5.39e-04

4.6 1 megabase Window

In the barplot that follows we examine the association of insertion sites with expression density in a 1 megabase window surrounding each locus. First, we count just the number of genes represented on the chip.

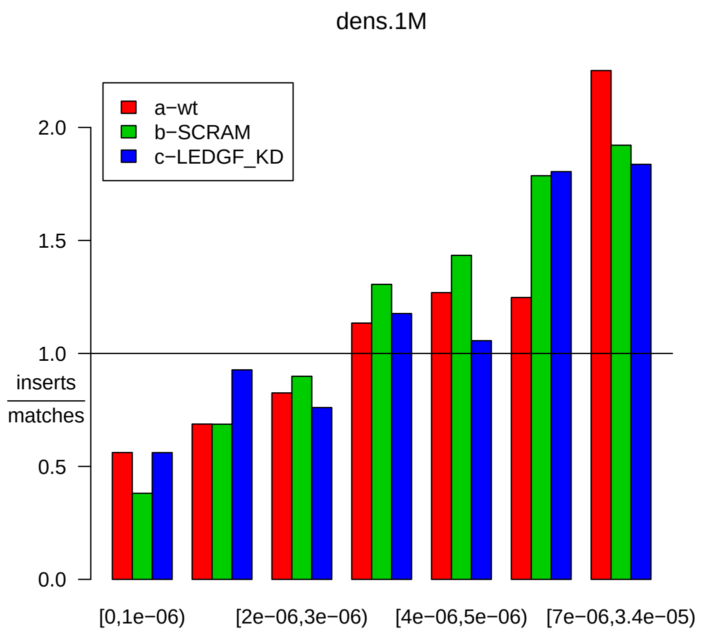

	coef	se	z	p
a-wt	0.824	0.0773	10.70	1.53e-26
b-SCRAM	0.946	0.0746	12.70	8.01e-37
c-LEDGF_KD	0.711	0.1720	4.14	3.46e-05

Here are the results for expression density. First, we count just genes that are in the upper half.

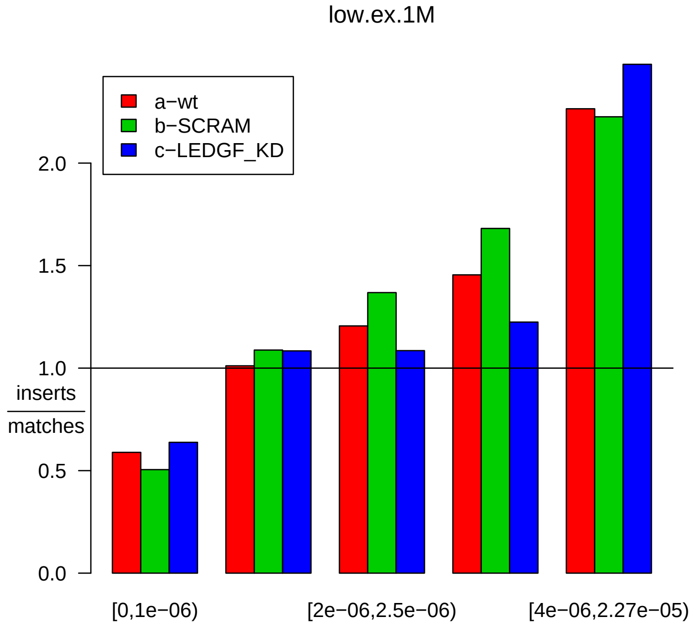


```
             coef     se    z        p
a-wt        0.913 0.0762 12.0 4.34e-33
b-SCRAM     0.997 0.0728 13.7 9.90e-43
c-LEDGF_KD  0.792 0.1690  4.7 2.60e-06
```

Now we count genes in the upper $1/8^{th}$:

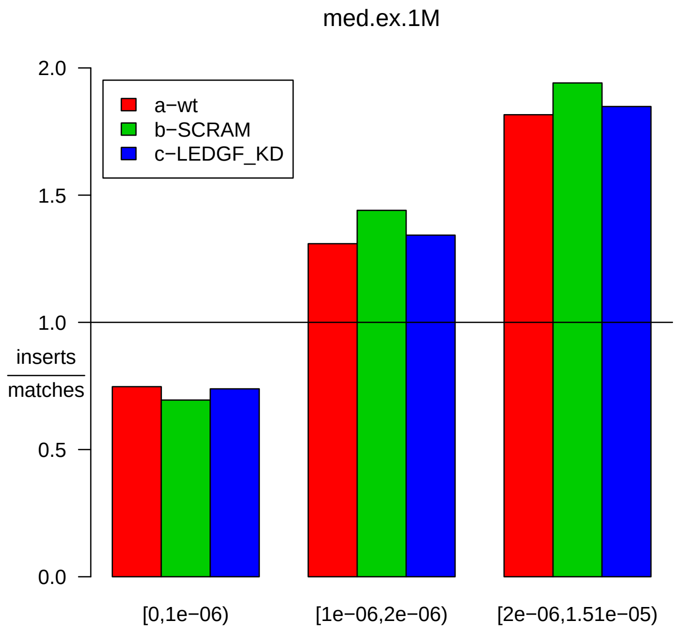

	coef	se	z	p
a-wt	0.782	0.0761	10.30	8.70e-25
b-SCRAM	0.940	0.0734	12.80	1.50e-37
c-LEDGF_KD	0.816	0.1710	4.76	1.91e-06

And here we count genes in the upper $1/16^{th}$:

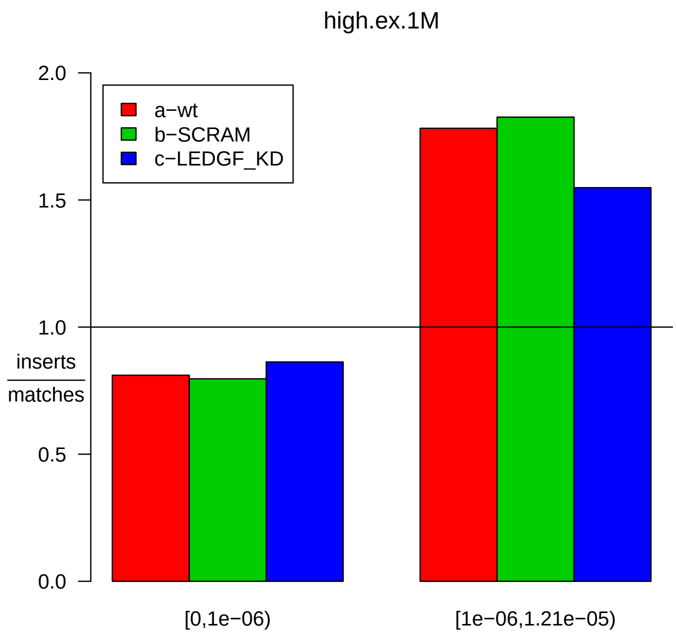

	coef	se	z	p
a-wt	0.870	0.0783	11.10	1.06e-28
b-SCRAM	0.811	0.0742	10.90	8.13e-28
c-LEDGF_KD	0.536	0.1800	2.98	2.90e-03

Here the effect of density of CpG islands is studied:

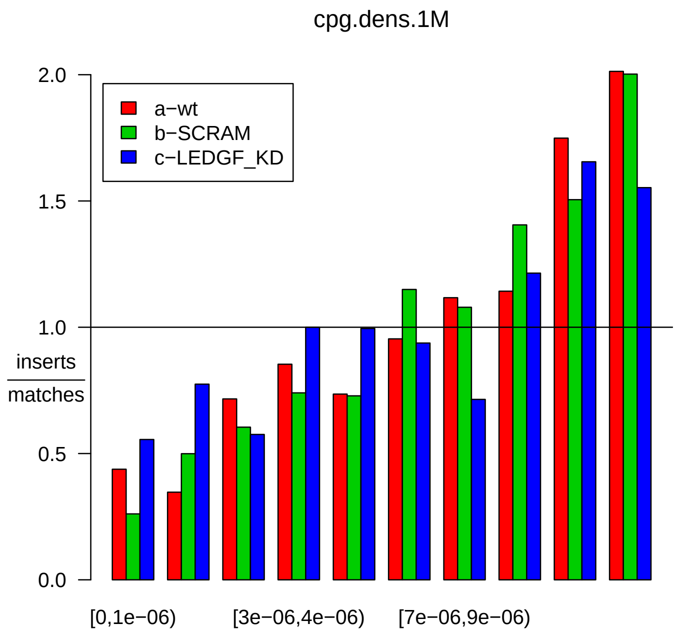

	coef	se	z	p
a-wt	0.825	0.0792	10.40	2.02e-25
b-SCRAM	0.873	0.0761	11.50	1.92e-30
c-LEDGF_KD	0.329	0.1680	1.96	5.04e-02

4.7 2 megabase Window

In the barplot that follows we examine the association of insertion sites with expression density in a 2 megabase window surrounding each locus. First, we count just the number of genes represented on the chip.

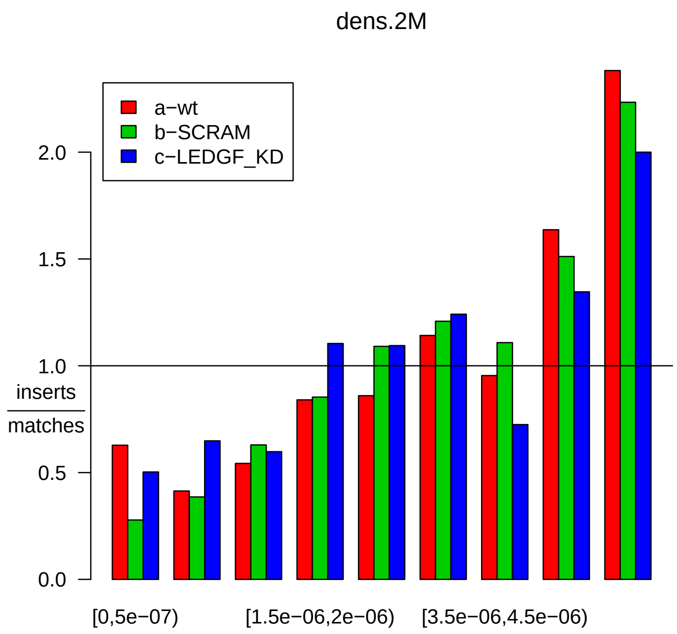

	coef	se	z	p
a-wt	0.782	0.0796	9.83	8.42e-23
b-SCRAM	0.870	0.0764	11.40	5.08e-30
c-LEDGF_KD	0.531	0.1720	3.10	1.96e-03

Here are the results for expression density. First, we count just genes that are in the upper half.

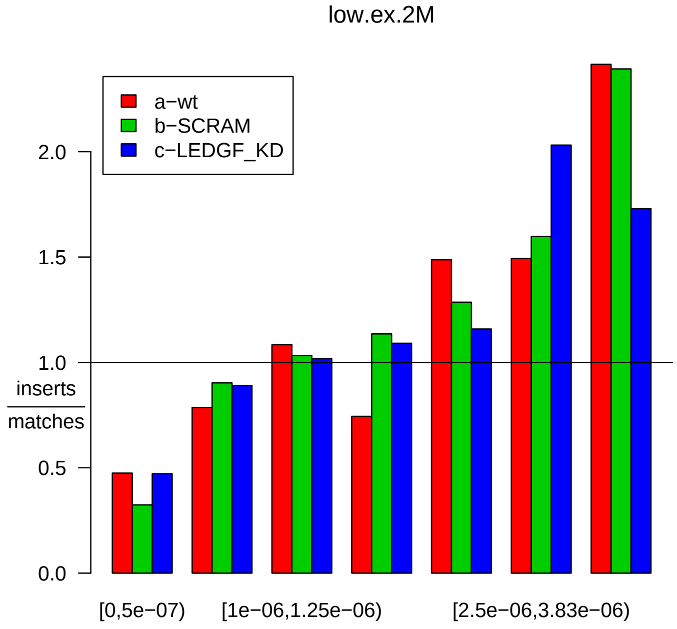

	coef	se	z	p
a-wt	0.817	0.0770	10.60	2.49e-26
b-SCRAM	0.884	0.0737	12.00	3.80e-33
c-LEDGF_KD	0.723	0.1700	4.26	2.06e-05

Now we count genes in the upper $1/8^{th}$:

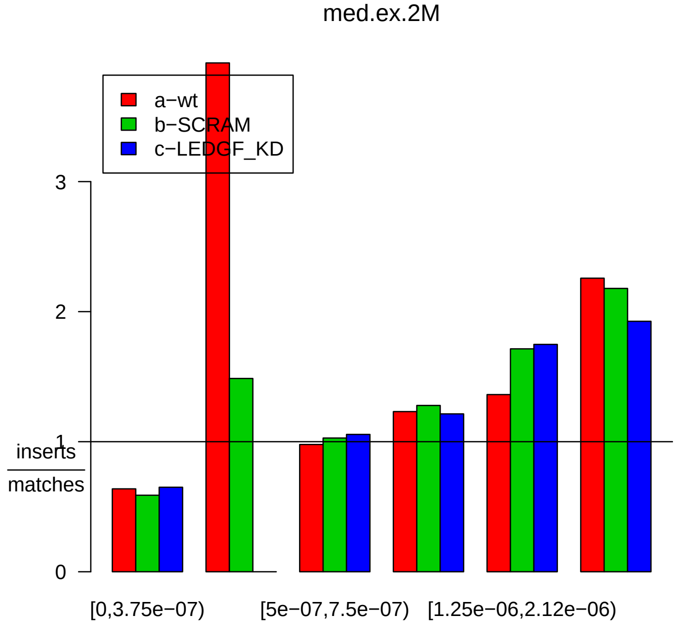

	coef	se	z	p
a-wt	0.746	0.0788	9.47	2.83e-21
b-SCRAM	0.869	0.0769	11.30	1.36e-29
c-LEDGF_KD	0.757	0.1800	4.21	2.57e-05

And here we count genes in the upper $1/16^{th}$:

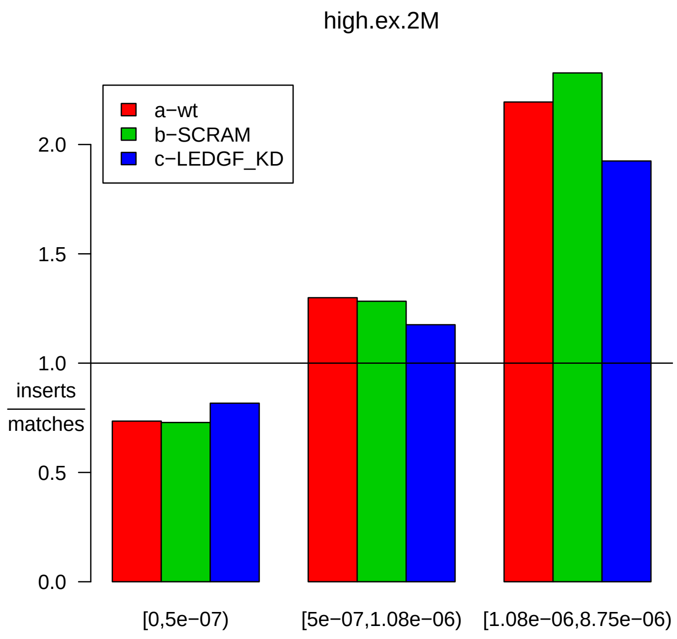

	coef	se	z	p
a-wt	0.790	0.0759	10.40	2.35e-25
b-SCRAM	0.875	0.0732	12.00	6.23e-33
c-LEDGF_KD	0.459	0.1690	2.72	6.62e-03

Here the effect of density of CpG islands is studied:

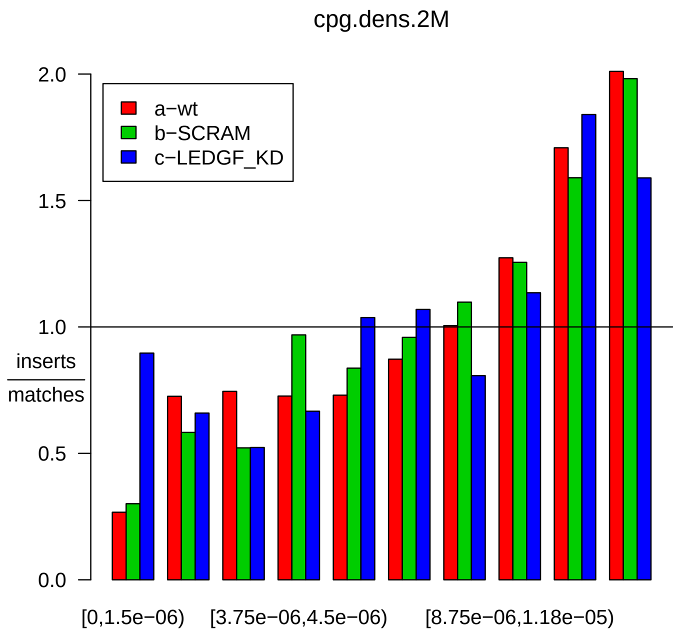

	coef	se	z	p
a-wt	0.714	0.0786	9.08	1.08e-19
b-SCRAM	0.731	0.0754	9.70	2.93e-22
c-LEDGF_KD	0.577	0.1730	3.33	8.77e-04

4.8 4 megabase Window

In the barplot that follows we examine the association of insertion sites with expression density in a 4 megabase window surrounding each locus. First, we count just the number of genes represented on the chip.

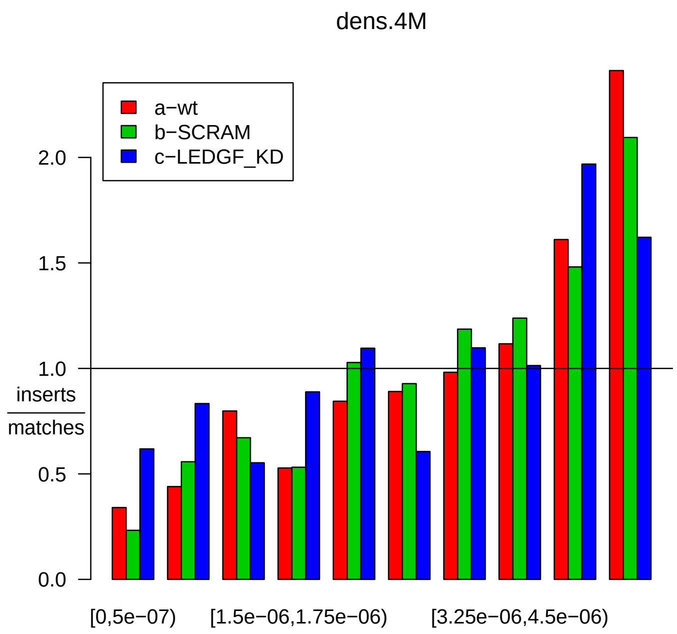

	coef	se	z	p
a-wt	0.770	0.0783	9.83	8.15e-23
b-SCRAM	0.739	0.0739	10.00	1.58e-23
c-LEDGF_KD	0.480	0.1700	2.82	4.81e-03

Here are the results for expression density. First, we count just genes that are in the upper half.

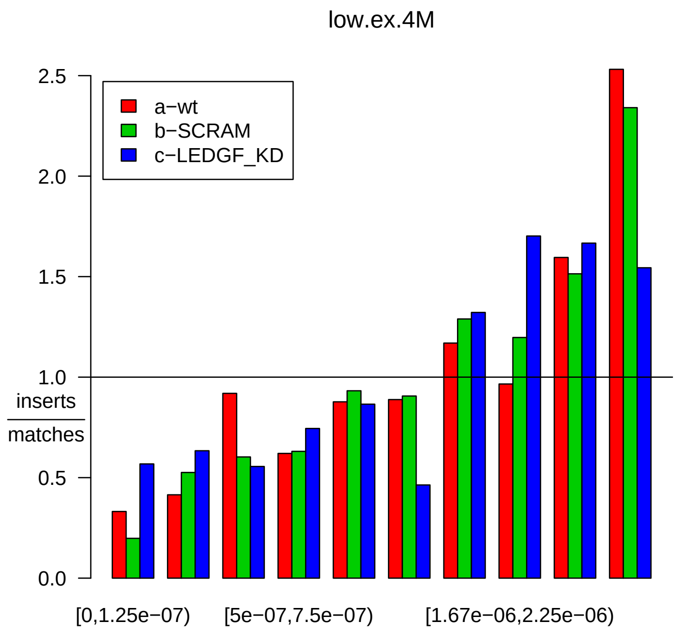

	coef	se	z	p
a-wt	0.843	0.0786	10.70	8.05e-27
b-SCRAM	0.851	0.0747	11.40	4.83e-30
c-LEDGF_KD	0.772	0.1750	4.41	1.02e-05

Now we count genes in the upper $1/8^{th}$:

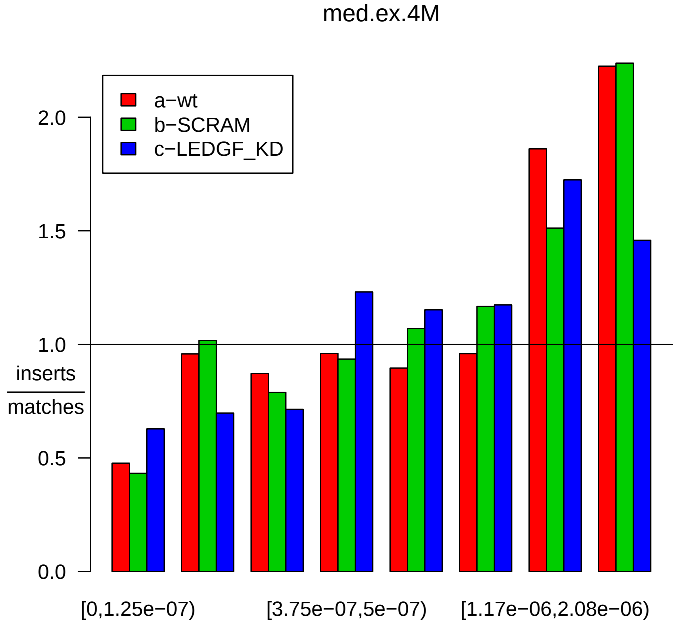

	coef	se	z	p
a-wt	0.752	0.0787	9.55	1.26e-21
b-SCRAM	0.805	0.0755	10.70	1.63e-26
c-LEDGF_KD	0.660	0.1750	3.77	1.64e-04

And here we count genes in the upper $1/16^{th}$:

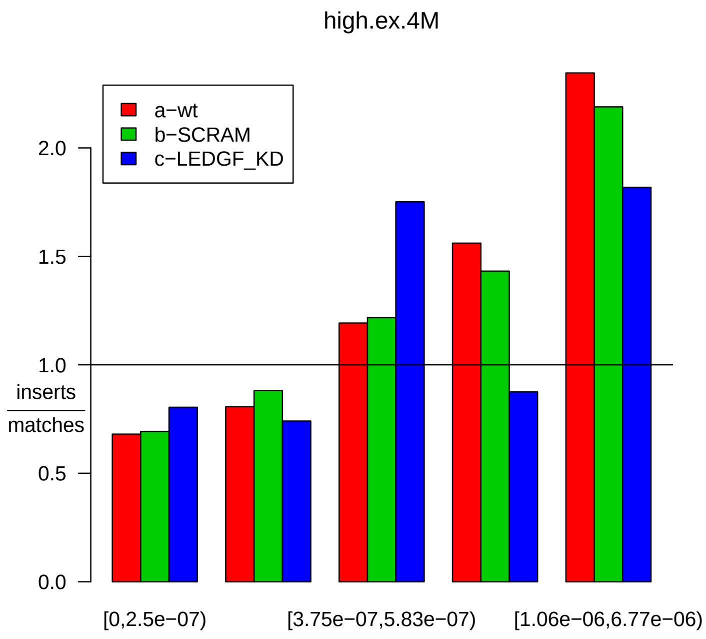

	coef	se	z	p
a-wt	0.872	0.0759	11.50	1.55e-30
b-SCRAM	0.700	0.0720	9.72	2.38e-22
c-LEDGF_KD	0.602	0.1710	3.53	4.14e-04

Here the effect of density of CpG islands is studied:

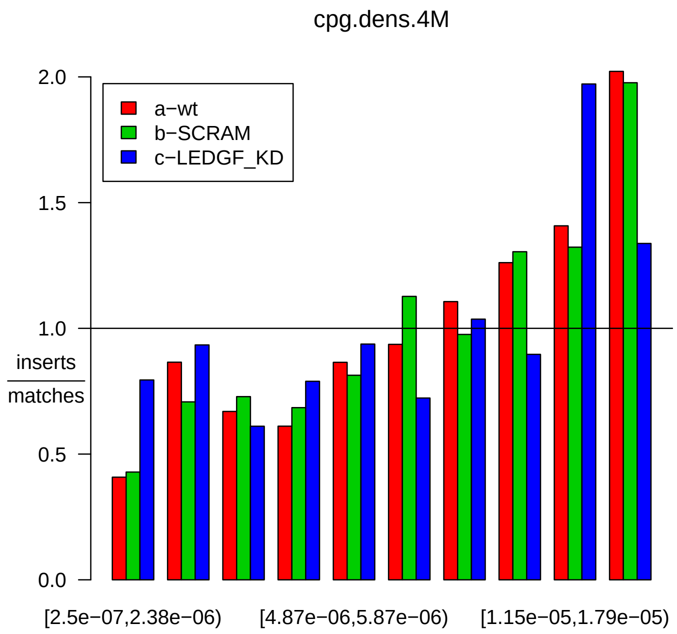

	coef	se	z	p
a-wt	0.681	0.0783	8.70	3.40e-18
b-SCRAM	0.673	0.0747	9.01	2.12e-19
c-LEDGF_KD	0.341	0.1700	2.01	4.45e-02

4.9 8 megabase Window

In the barplot that follows we examine the association of insertion sites with expression density in a 8 megabase window surrounding each locus. First, we count just the number of genes represented on the chip.

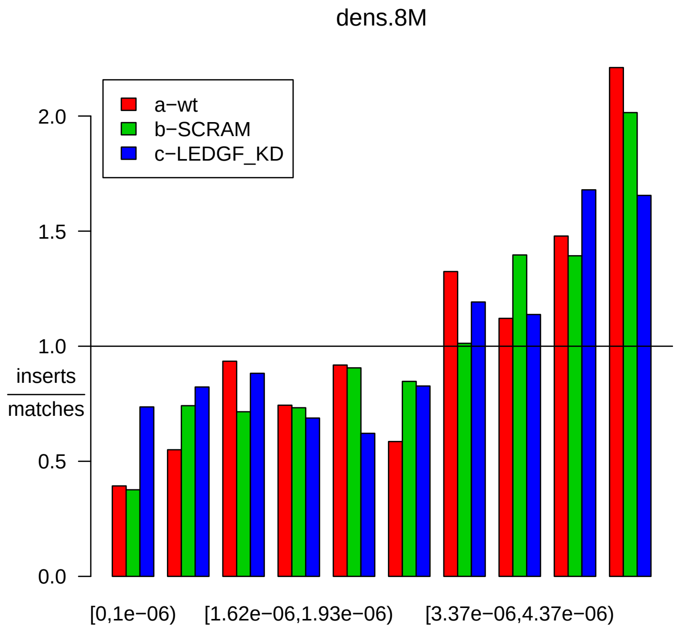

	coef	se	z	p
a-wt	0.643	0.0779	8.26	1.52e-16
b-SCRAM	0.656	0.0743	8.83	1.07e-18
c-LEDGF_KD	0.564	0.1710	3.29	9.95e-04

Here are the results for expression density. First, we count just genes that are in the upper half.

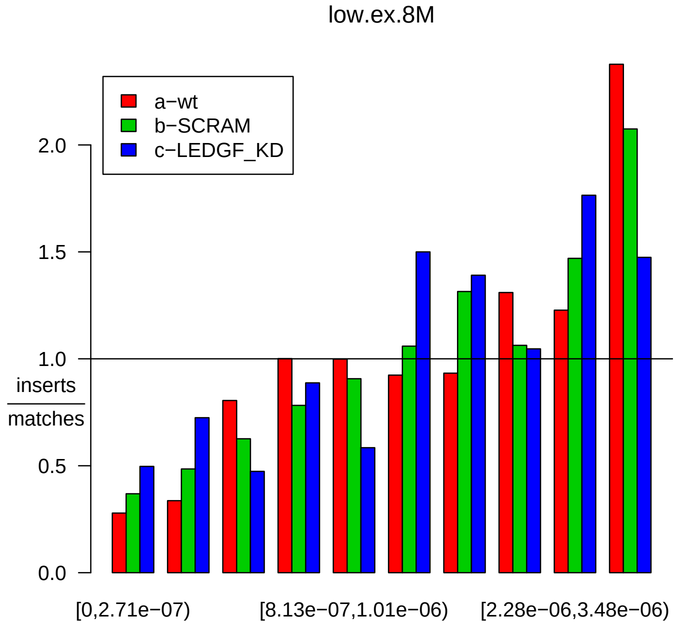

	coef	se	z	p
a-wt	0.662	0.0783	8.46	2.71e-17
b-SCRAM	0.786	0.0759	10.40	3.62e-25
c-LEDGF_KD	0.809	0.1780	4.54	5.74e-06

Now we count genes in the upper $1/8^{th}$:

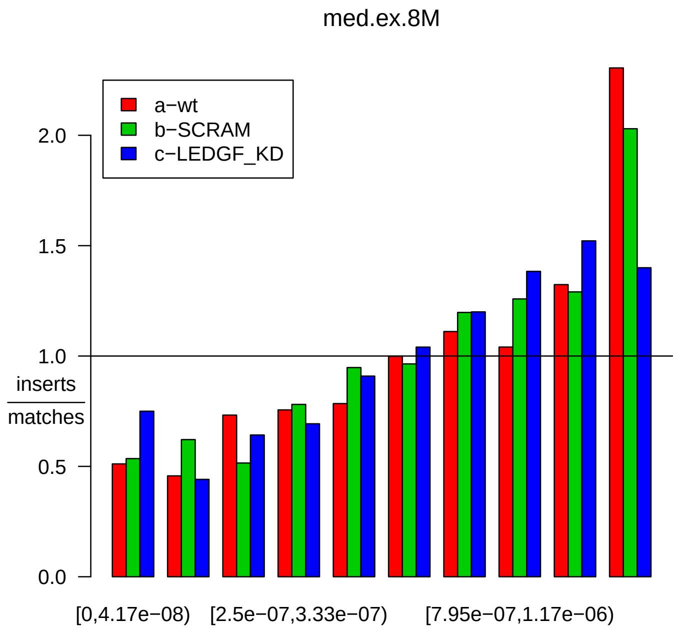

	coef	se	z	p
a-wt	0.708	0.0788	8.98	2.76e-19
b-SCRAM	0.688	0.0745	9.23	2.73e-20
c-LEDGF_KD	0.618	0.1750	3.54	4.02e-04

And here we count genes in the upper $1/16^{th}$:

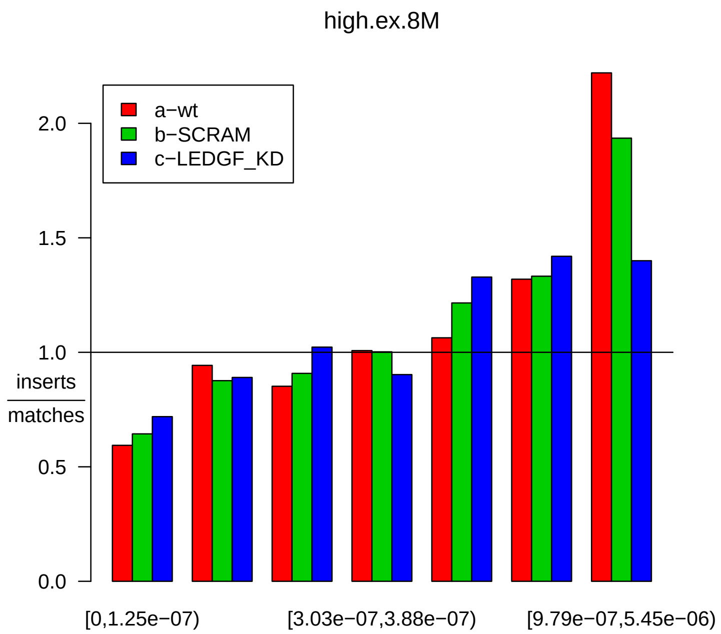

	coef	se	z	p
a-wt	0.636	0.0759	8.38	5.47e-17
b-SCRAM	0.560	0.0720	7.78	7.00e-15
c-LEDGF_KD	0.485	0.1680	2.88	3.97e-03

Here the effect of density of CpG islands is studied:

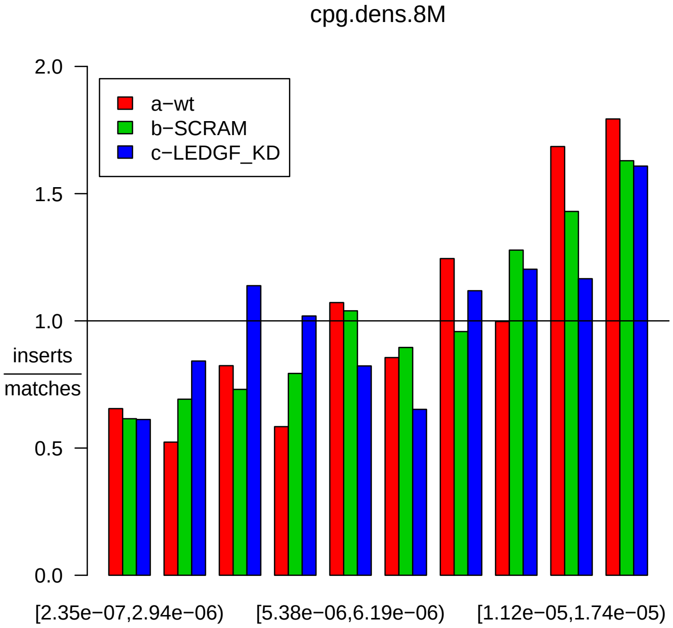

	coef	se	z	p
a-wt	0.608	0.0777	7.83	5.03e-15
b-SCRAM	0.486	0.0731	6.64	3.04e-11
c-LEDGF_KD	0.274	0.1700	1.61	1.08e-01

4.10 16 megabase Window

In the barplot that follows we examine the association of insertion sites with expression density in a 16 megabase window surrounding each locus. First, we count just the number of genes represented on the chip.

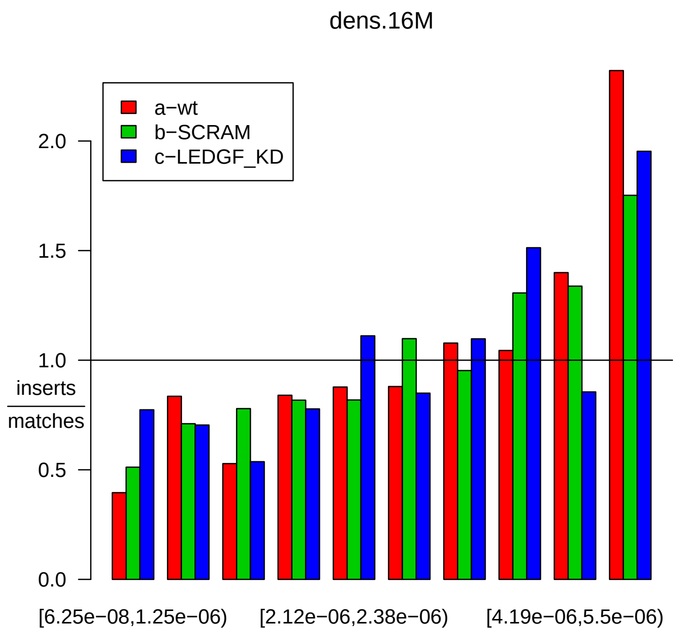

	coef	se	z	p
a-wt	0.650	0.0781	8.32	8.45e-17
b-SCRAM	0.572	0.0736	7.77	7.68e-15
c-LEDGF_KD	0.452	0.1720	2.63	8.66e-03

Here are the results for expression density. First, we count just genes that are in the upper half.

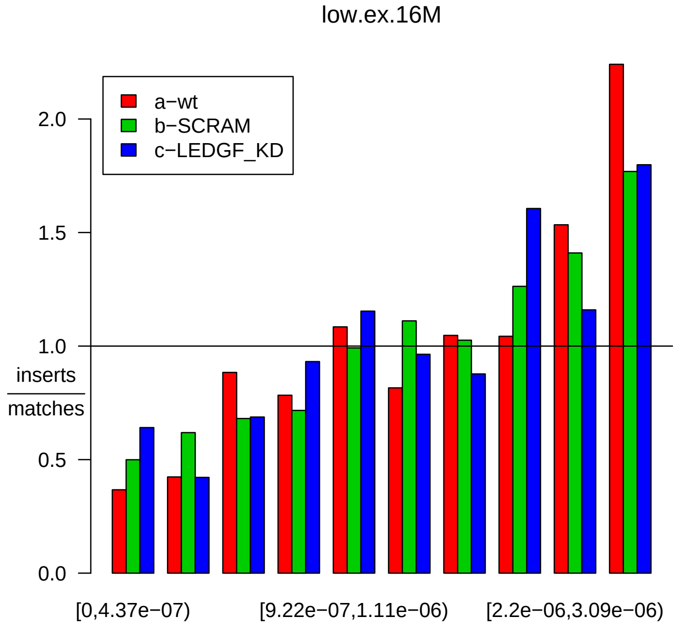


```
             coef     se    z         p
a-wt        0.638 0.0778 8.20 2.33e-16
b-SCRAM     0.626 0.0742 8.43 3.40e-17
c-LEDGF_KD  0.497 0.1730 2.88 4.03e-03
```

Now we count genes in the upper $1/8^{th}$:

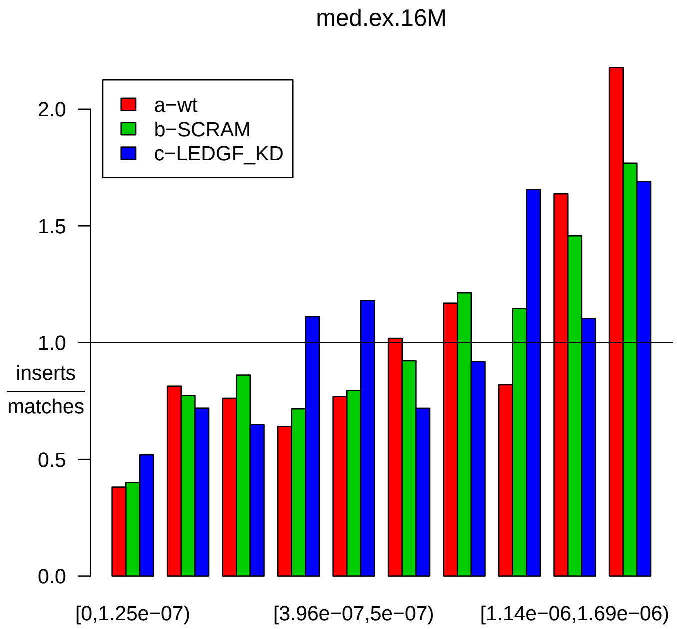

	coef	se	z	p
a-wt	0.656	0.0779	8.43	3.45e-17
b-SCRAM	0.545	0.0735	7.41	1.23e-13
c-LEDGF_KD	0.369	0.1700	2.17	3.03e-02

And here we count genes in the upper $1/16^{th}$:

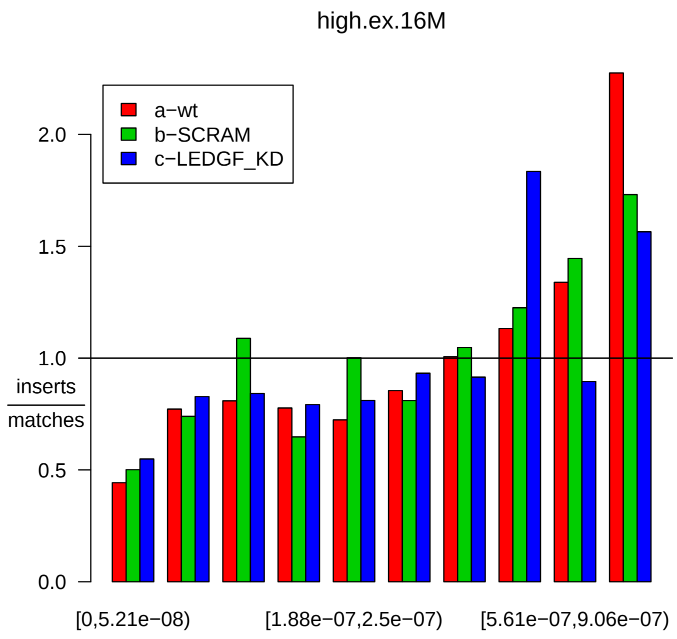


```
            coef     se    z        p
a-wt       0.608 0.0776 7.84 4.50e-15
b-SCRAM    0.503 0.0730 6.89 5.49e-12
c-LEDGF_KD 0.444 0.1700 2.62 8.89e-03
```

Here the effect of density of CpG islands is studied:

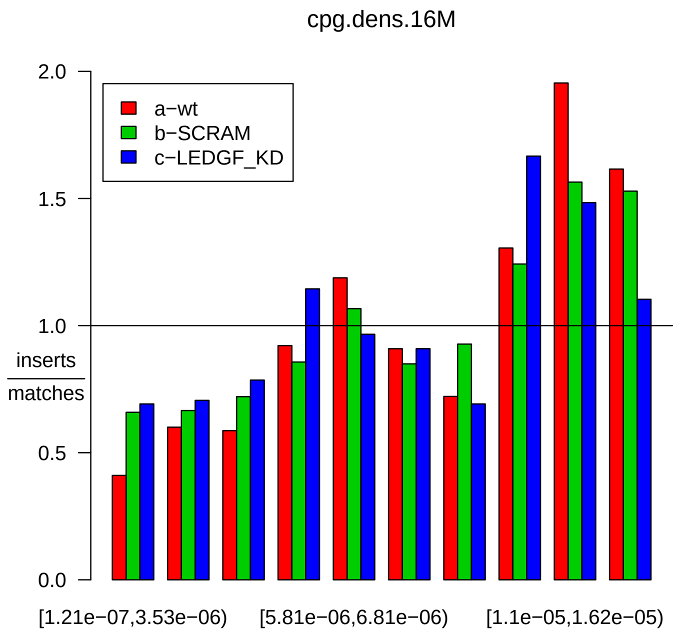


```
            coef     se    z        p
a-wt       0.566 0.0776 7.30 2.98e-13
b-SCRAM    0.436 0.0728 5.99 2.12e-09
c-LEDGF_KD 0.282 0.1690 1.67 9.54e-02
```

4.11 32 megabase Window

In the barplot that follows we examine the association of insertion sites with expression density in a 32 megabase window surrounding each locus. First, we count just the number of genes represented on the chip.

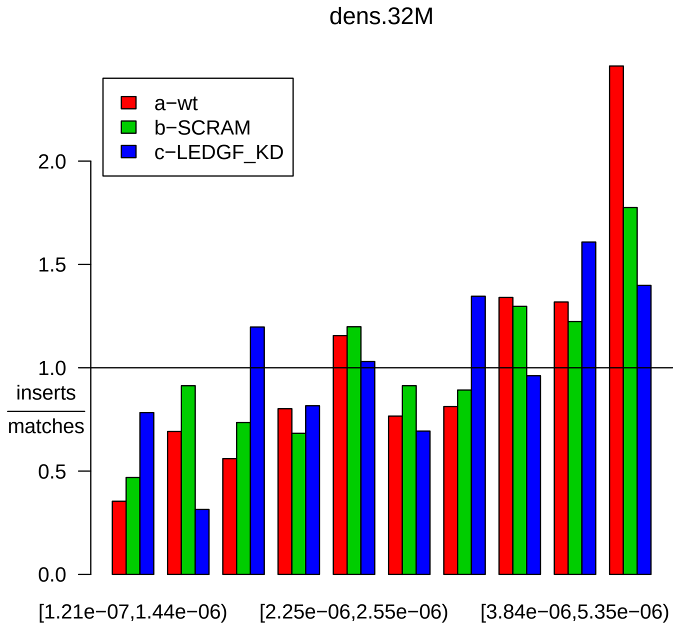

	coef	se	z	p
a-wt	0.618	0.0778	7.95	1.89e-15
b-SCRAM	0.427	0.0727	5.87	4.42e-09
c-LEDGF_KD	0.365	0.1720	2.13	3.34e-02

Here are the results for expression density. First, we count just genes that are in the upper half.

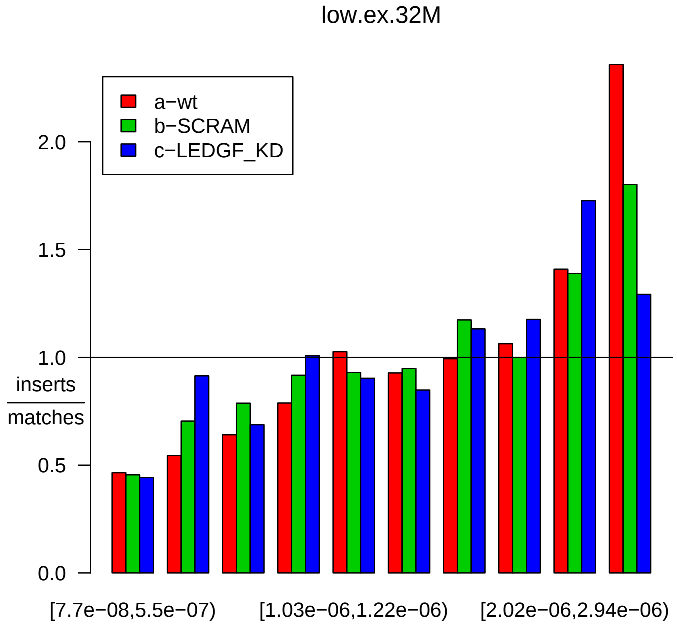

	coef	se	z	p
a-wt	0.667	0.0784	8.51	1.80e-17
b-SCRAM	0.511	0.0732	6.97	3.10e-12
c-LEDGF_KD	0.436	0.1720	2.54	1.11e-02

Now we count genes in the upper $1/8^{th}$:

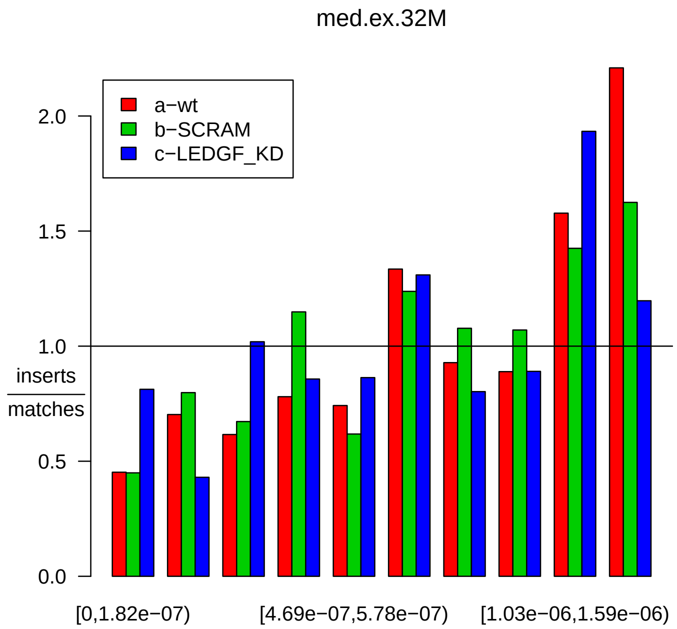

	coef	se	z	p
a-wt	0.732	0.0791	9.26	2.09e-20
b-SCRAM	0.567	0.0737	7.70	1.38e-14
c-LEDGF_KD	0.450	0.1720	2.62	8.89e-03

And here we count genes in the upper $1/16^{th}$:

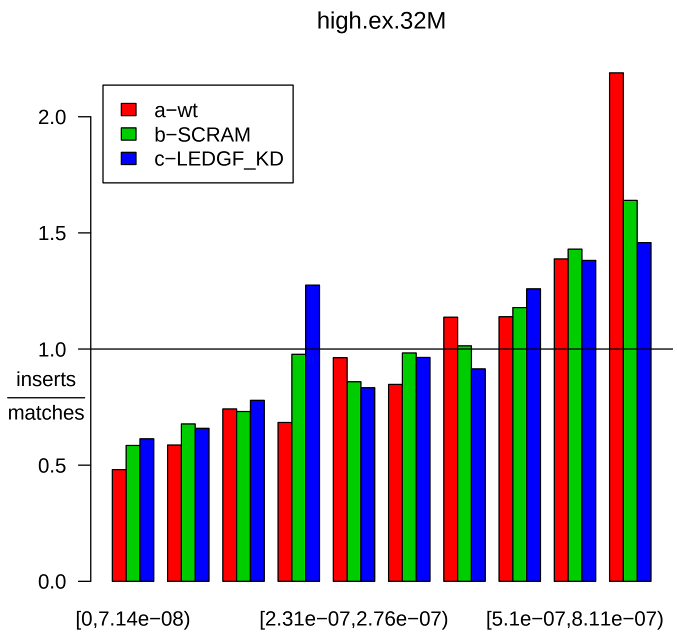

	coef	se	z	p
a-wt	0.671	0.0785	8.55	1.27e-17
b-SCRAM	0.505	0.0729	6.92	4.38e-12
c-LEDGF_KD	0.372	0.1690	2.20	2.81e-02

Here the effect of density of CpG islands is studied:

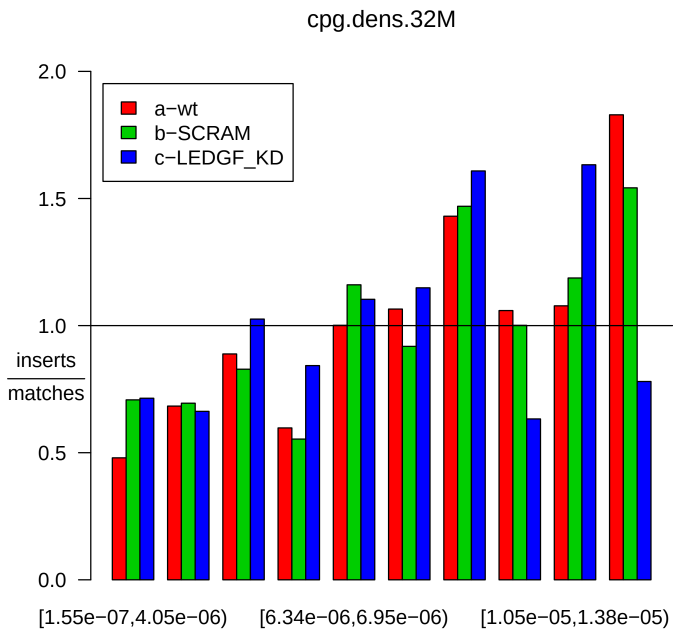

	coef	se	z	p
a-wt	0.563	0.0774	7.27	3.56e-13
b-SCRAM	0.429	0.0726	5.90	3.59e-09
c-LEDGF_KD	0.297	0.1690	1.75	7.93e-02

5 Juxtaposition with Gene Start and End Positions

5.1 Acembly Annotations

In this section we study the effect of juxtaposition in terms of gene start and end positions. The first barplot shows the effect of gene width for those insertions that are located within an Acembly gene. The table following the barplot shows the p-values for a test of the hypothesis that the proportions in each of the categories that define the bars are equal in the insertions and their matches. This p-value is obtained from the $5 \times 2 \times k$ table of counts defined by gene width category, insertion/match status, and stratum (consisting of an insertion and its matched sites) using a likelihood ratio test for the hypothesis of no association between gene width category and insertion/match status. The test used compared the log-linear model [1] with all two-way configurations to that with no gene width category and insertion/match status configuration.

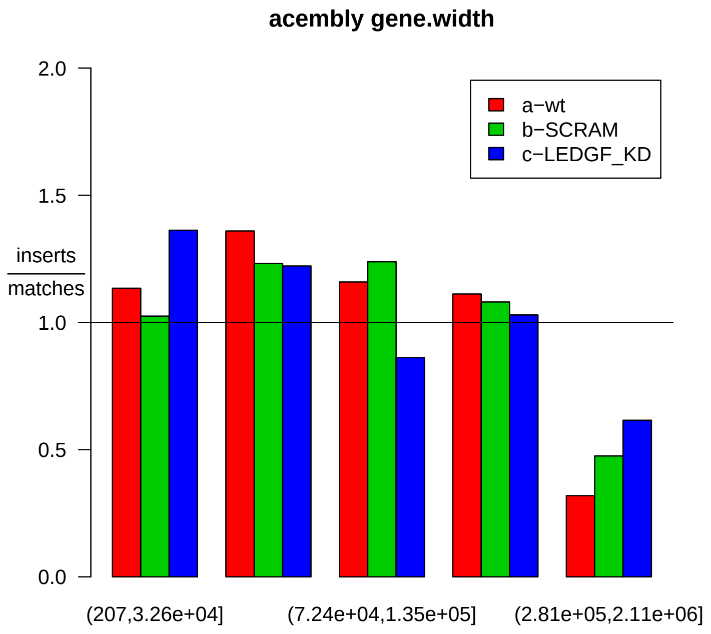

a-wt	b-SCRAM	c-LEDGF_KD
1.34e-19	6.20e-13	8.54e-02

The next plot uses the width of a non-gene region for insertions that fall into such regions.

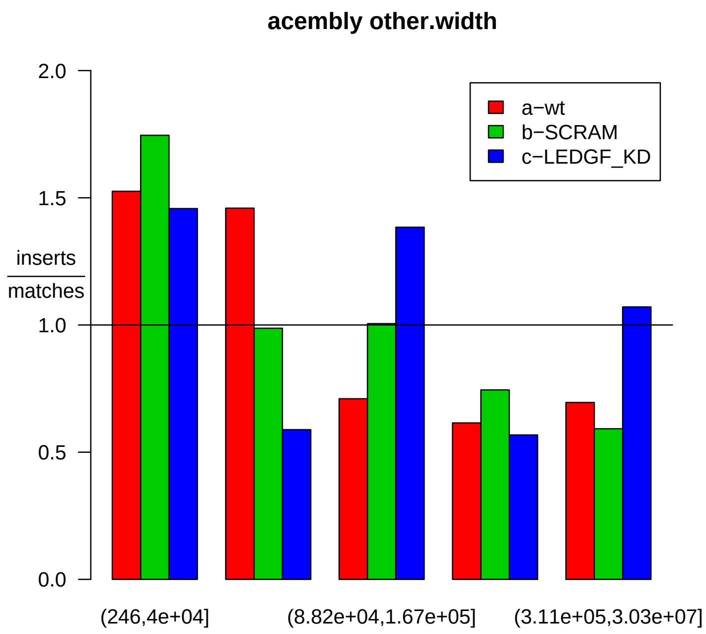


```
     a-wt    b-SCRAM c-LEDGF_KD
 5.34e-07   9.93e-06   4.08e-02
```

The next plot studies the distance to the nearest boundary between a gene and a non-gene region. The distance is expressed as a fraction of the length of the region. Thus, '0.25' refers to one quarter of the distance from the site to nearest boundary divided by the total width of the region.

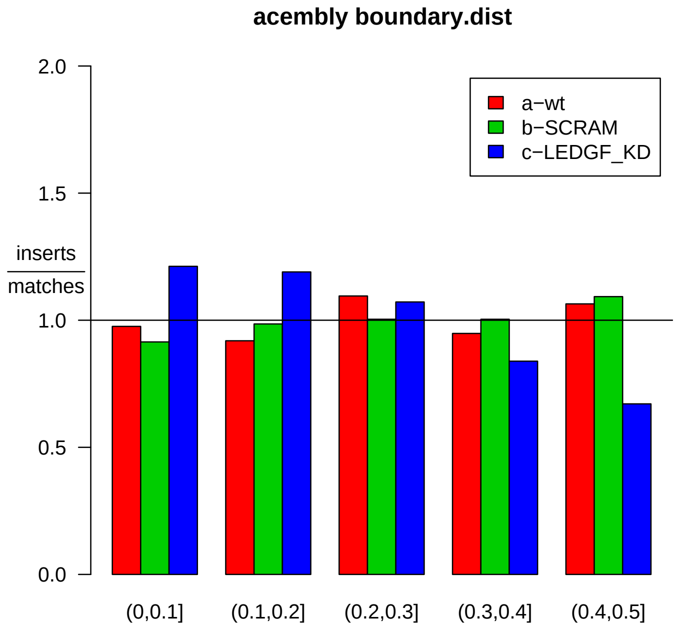

a-wt	b-SCRAM	c-LEDGF_KD
0.475	0.580	0.126

This plot studies the effect of nearness to the beginning of a transcript. For sites in genes, it is the distance to the start of the gene divided by the width of the gene. For other sites it is the distance from the site to the nearer gene if that gene boundary is also a transcription starting point. Locations near '0' are relatively near the beginning of transcription, while those near '1' are near the termination of the transcript.

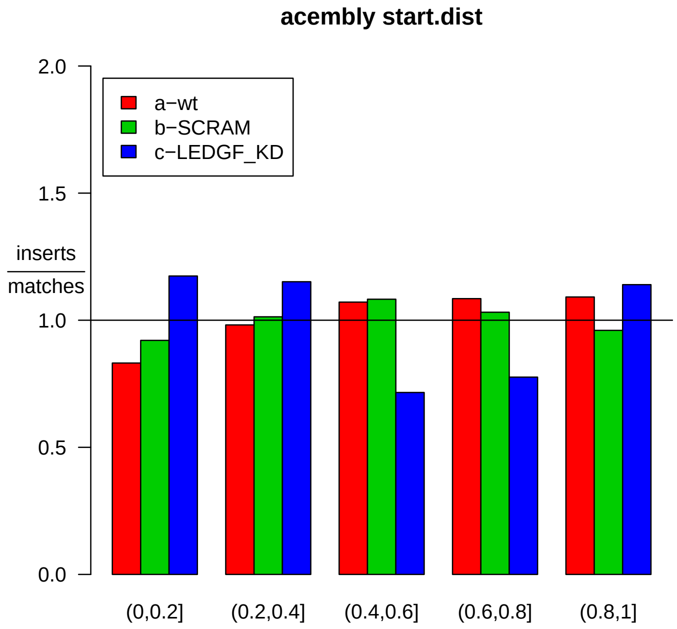

a-wt	b-SCRAM	c-LEDGF_KD
0.0792	0.5350	0.1960

5.2 RefSeq Annotations

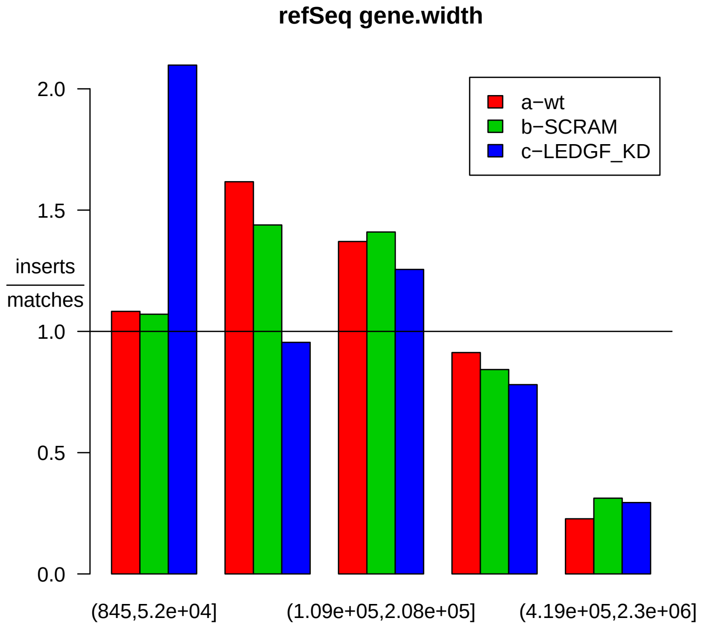

a-wt	b-SCRAM	c-LEDGF_KD
1.08e-24	5.07e-22	1.74e-04

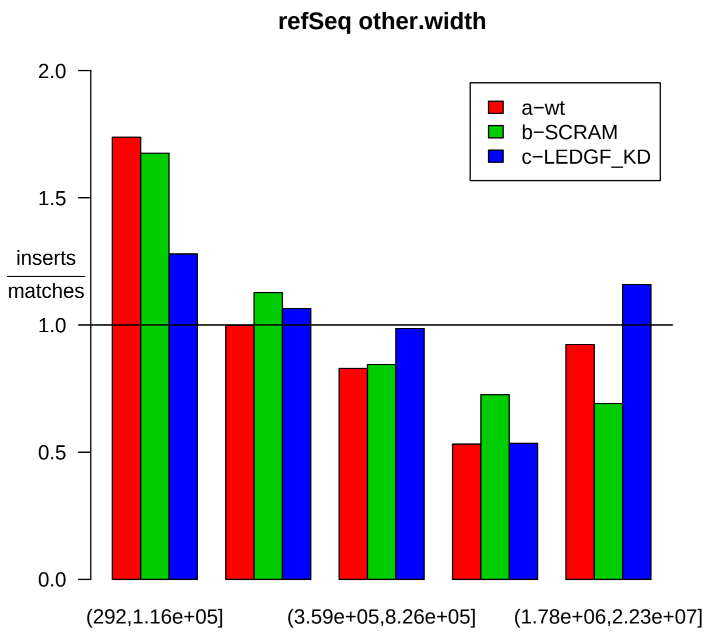

a-wt	b-SCRAM	c-LEDGF_KD
2.95e-10	2.42e-07	1.81e-01

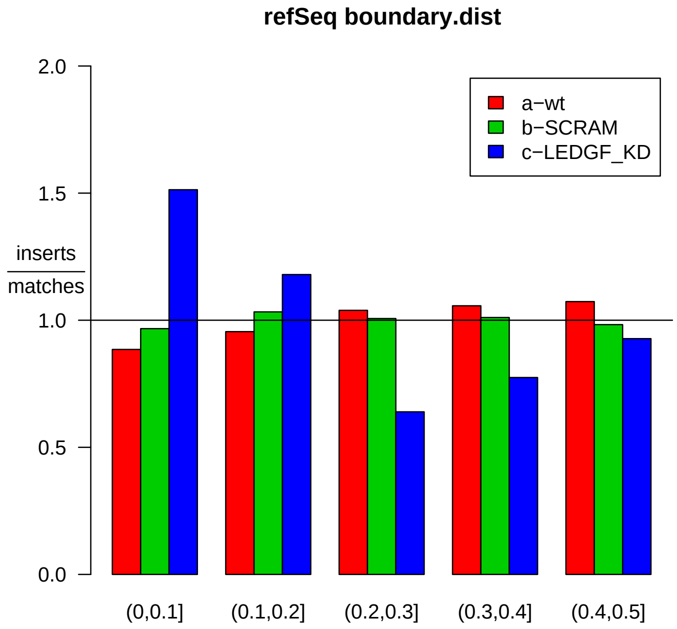

a-wt	b-SCRAM	c-LEDGF_KD
0.38500	0.98200	0.00653

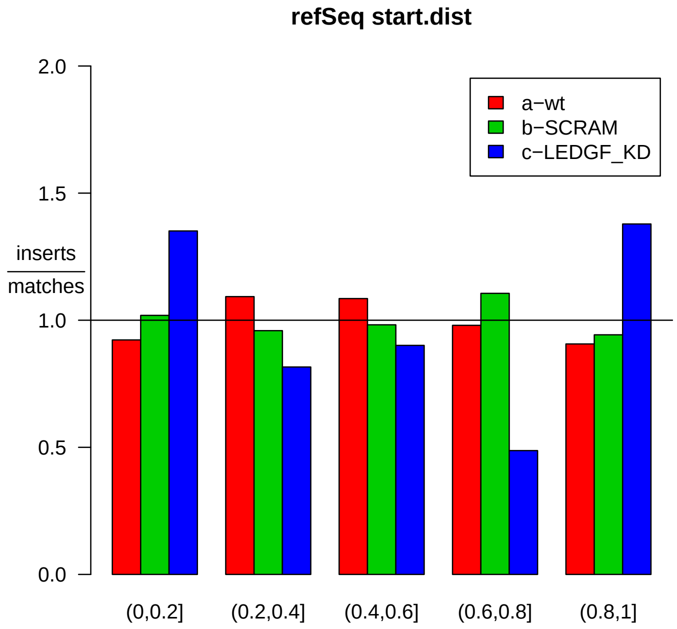

a-wt	b-SCRAM	c-LEDGF_KD
0.35500	0.59400	0.00458

5.3 genScan Annotations

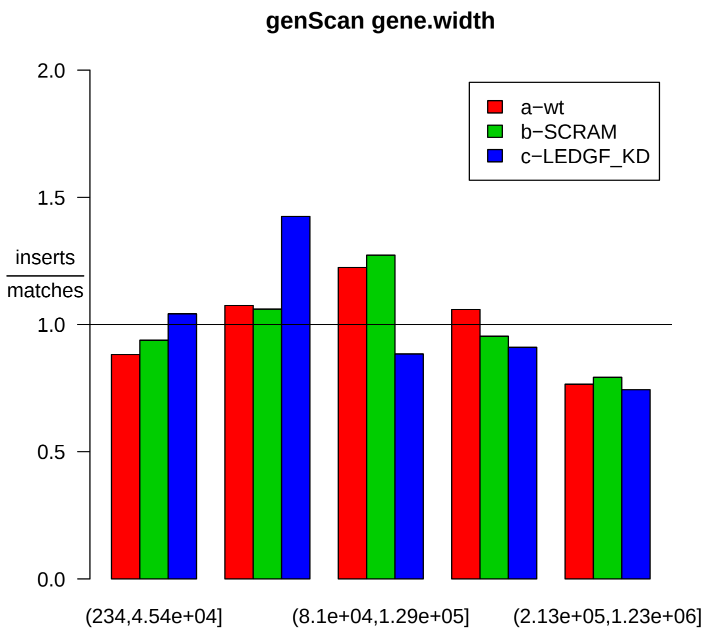


```
    a-wt     b-SCRAM  c-LEDGF_KD
 0.00565     0.00586     0.26600
```

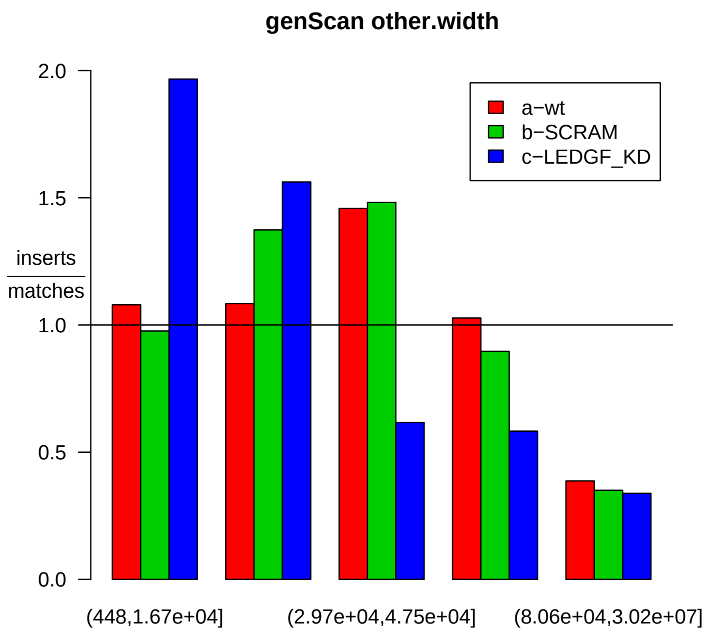

a-wt	b-SCRAM	c-LEDGF_KD
6.89e-05	2.47e-07	3.84e-03

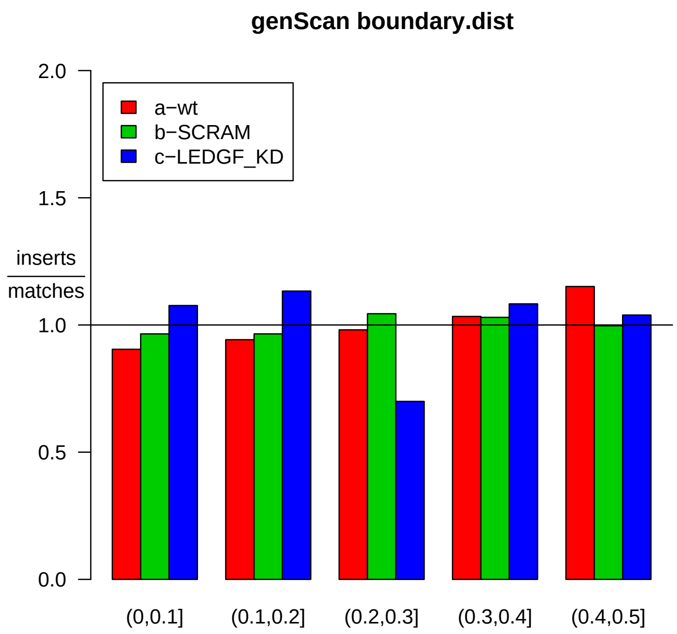

a-wt	b-SCRAM	c-LEDGF_KD
0.246	0.921	0.365

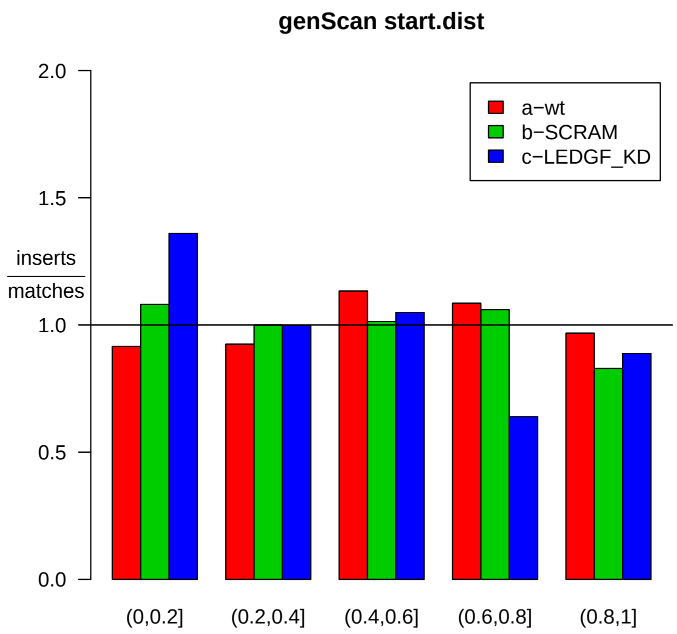

a-wt	b-SCRAM	c-LEDGF_KD
0.2430	0.2310	0.0476

5.4 uniGene Annotations

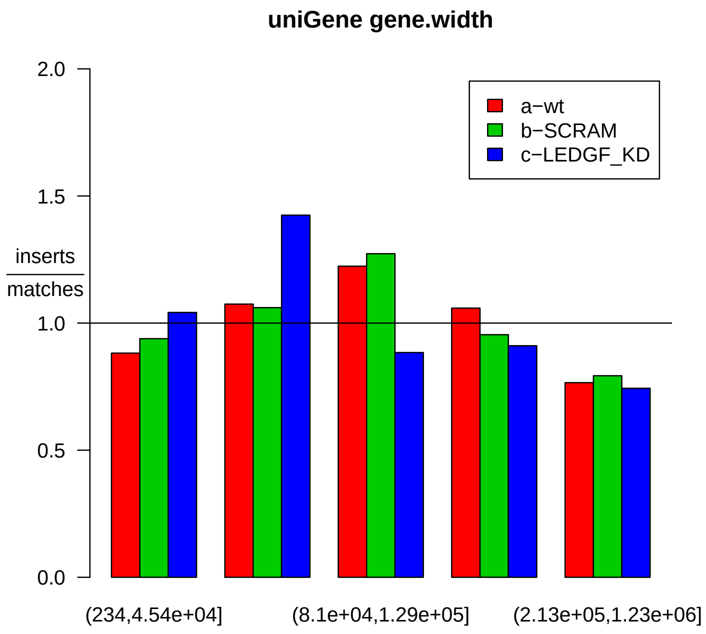


```
   a-wt    b-SCRAM c-LEDGF_KD
0.00565    0.00586    0.26600
```

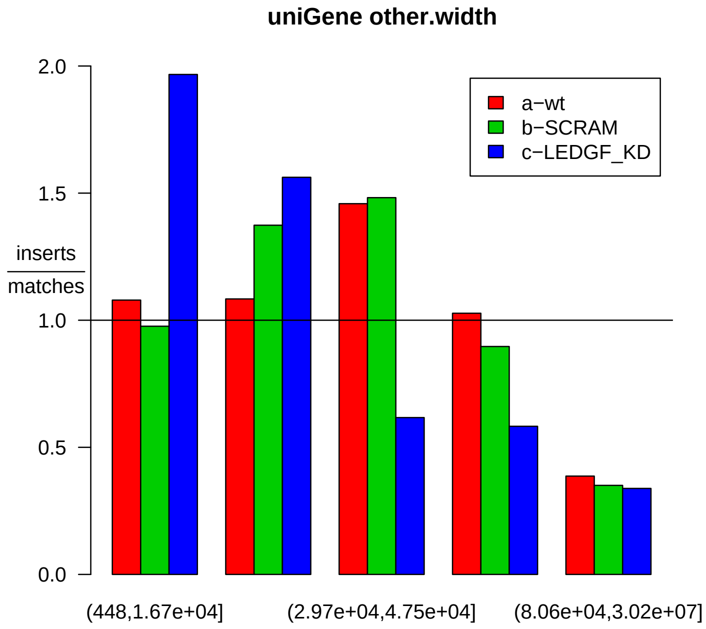

a-wt	b-SCRAM	c-LEDGF_KD
6.89e-05	2.47e-07	3.84e-03

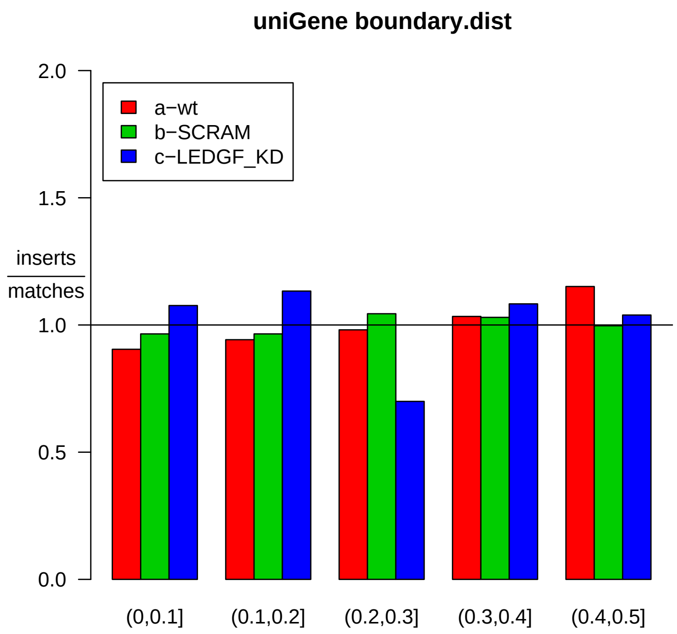

a-wt	b-SCRAM	c-LEDGF_KD
0.246	0.921	0.365

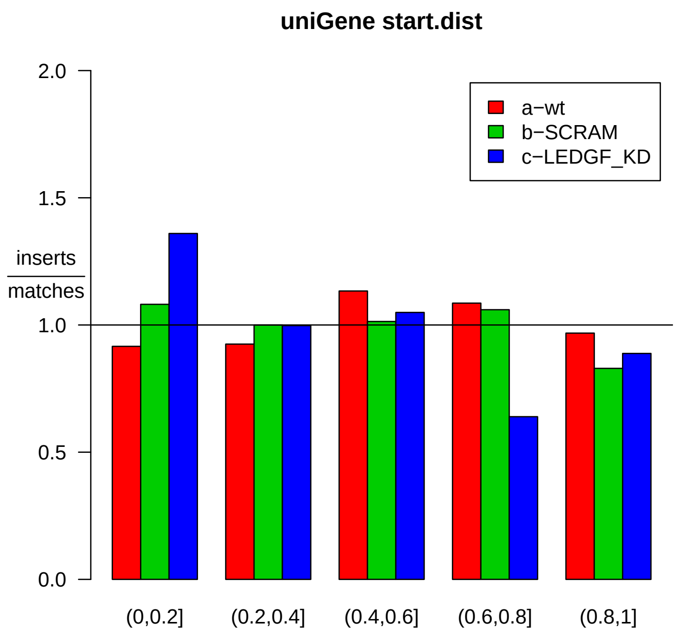


```
  a-wt    b-SCRAM c-LEDGF_KD
0.2430     0.2310     0.0476
```

6 GC content

Here we study the effect of GC content on insertion. The GC content is taken from the Human Genome Draft at GoldenPath from the table `http://genome.ucsc.edu/goldenPath/hg17/database/gc5Base.txt.gz`.

Following the plot is a table of fitted coefficients based on splitting the GC percent data at the median.

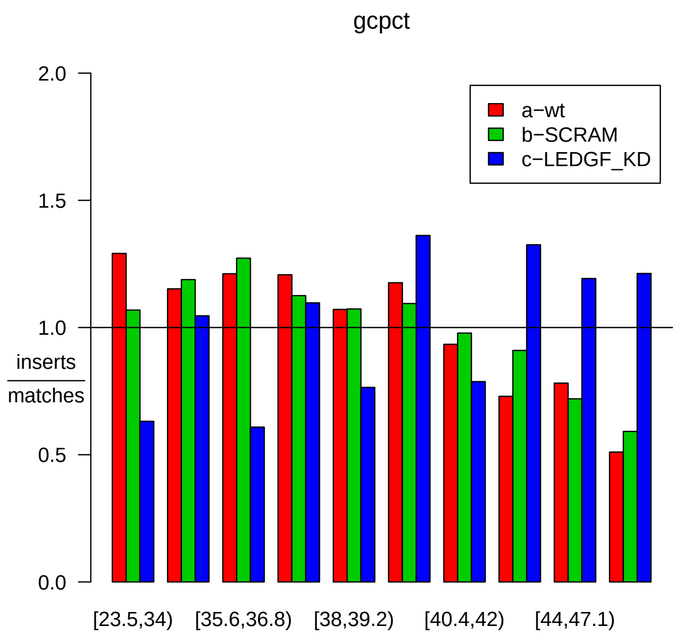

	coef	se	z	p
a-wt	-0.379	0.0770	-4.93	8.38e-07
b-SCRAM	-0.294	0.0727	-4.05	5.15e-05
c-LEDGF_KD	0.327	0.1720	1.90	5.77e-02

7 Cytobands

Here we study the association of cytoband with insertion intensity. The data are obtained from
`http://genome.ucsc.edu/goldenPath/hg17/database/cytoBand.txt.gz`.

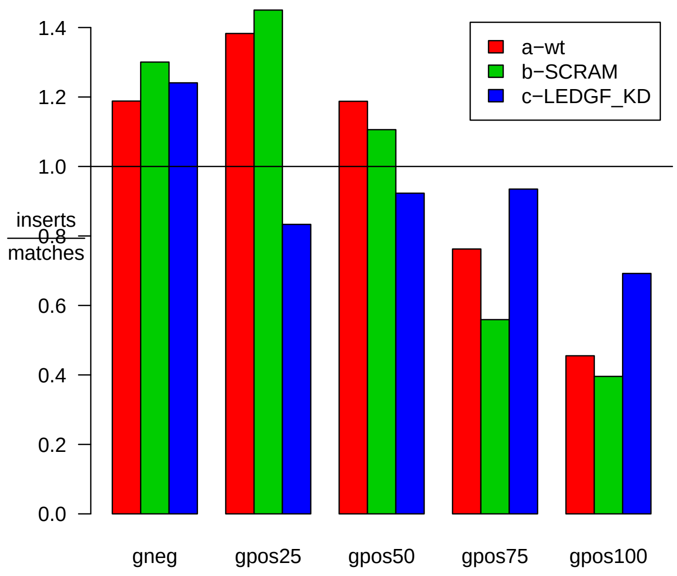

A formal test of significance attains a p-value of $< 2.22e - 16$. Here is the table of coefficients of the log ratio of intensities for true insertion sites versus control insertion sites (comparing each category of Giemsa staining to 'gneg') along with their standard errors, z statistics, and p-values:

	coef	se	z	p
cyto.typegpos100	-1.0300	0.0903	-11.40	5.23e-30
cyto.typegpos25	0.0971	0.0883	1.10	2.71e-01
cyto.typegpos50	-0.1030	0.0699	-1.47	1.42e-01
cyto.typegpos75	-0.6040	0.0842	-7.17	7.27e-13

References

[1] Yvonne M.M. Bishop, Stephen E. Fienberg, and Paul W. Holland. *Discrete multivariate analyses: Theory and practice* (MIT Press, 1975).

[2] P. McCullagh and John A. Nelder. *Generalized linear models.* (Chapman & Hall ltd, 1999).

[3] Xiaolin Wu, Yuan Li, Bruce Crise, Shawn M. Burgess "Transcription Start Regions in the Human Genome Are Favored Targets for MLV Integration," *Science,* **300**(5626), (June 2003): 1749-1751.
